# Thermoresistant flagellin-adjuvanted cancer vaccine combined with photothermal therapy synergizes with anti-PD-1 treatment

**DOI:** 10.1136/jitc-2024-010272

**Published:** 2025-03-20

**Authors:** Jayalakshmi Thiruppathi, Veena Vijayan, Hye Suk Hwang, Yong Jun Bang, Vandara Loeurng, Seol Hee Hong, Aravindkumar Sundaram, In-Kyu Park, Shee Eun Lee, Joon Haeng Rhee

**Affiliations:** 1 Chonnam National University, Hwasun, Korea (the Republic of); 2 Department of Biomedical Science,College of Life Science and Industry, Sunchon National University, Sunchon 57922, South Korea; 3 Chonnam National University, Gwangju, Korea (the Republic of); 4 Chonnam National University Medical School, Gwangju, Korea (the Republic of)

**Keywords:** Immune Checkpoint Inhibitor, Abscopal, Adjuvant, Breast Cancer, Vaccine

## Abstract

**Background:**

Cancer immunotherapy, leveraging the immune system to target and eradicate cancer cells, has transformed cancer treatment paradigms. Immune checkpoint inhibitors (ICIs) are used in a wide array of cancers, but only a limited fraction of patients are responding. Cancer vaccines could elicit antigen-specific immune responses and establish long-term immune memory, preventing recurrence and metastasis. Despite their promising profiles, ICIs and cancer vaccines by themselves are often insufficient to overcome the immunosuppressive tumor microenvironment (TME) and recurrence/metastasis. Addressing these challenges is crucial for improving cancer immunotherapy outcomes.

**Methods:**

The targeted liposomal formulation (TLIF), displaying Cyclic RGD (cRGD) peptide on the surface and encapsulating ICG and thermoresistant flagellin (FlaB) inside, was used for photothermal therapy (PTT), which was designed to induce robust immunogenic cell death (ICD) and release tumor antigens (TAs). We employed a mouse breast cancer model amenable to PTT. Utilizing a bilateral DD-Her2/neu tumor implantation model, we evaluated local and abscopal effects of combinatorial approaches employing PTT, FlaB-adjuvanted peptide vaccine (FlaB-Vax), and anti-PD-1 treatment. FlaB-Vax was designed to trigger tumor-associated antigen (TAA)-specific immune responses, which will trigger specific anti-tumor immunity. TLIF-PTT aimed to reduce tumor burden and induce ICD-mediated TA liberation for epitope spreading. Sustained anti-tumor immune memory was assessed by orthotopic rechallenging cured mice with the DD-Her2/neu tumor cells.

**Results:**

The combination of TLIF-PTT and FlaB-Vax provided significantly enhanced primary tumor suppression, with strong abscopal effects and long-lasting immune memory. The addition of anti-PD-1 therapy further improved long-term relapse-free survival, highlighting the potential of this combinatorial approach to induce durable antitumor immunity and sustainably prevent cancer recurrence and metastasis.

**Conclusion:**

This study demonstrates that the combination of TLIF-PTT and FlaB-Vax synergistically induced synergistic anti-tumor immune responses, which were efficaciously potentiated by anti-PD-1 treatment for recurrence-free long-term survival.

**WHAT IS ALREADY KNOWN ON THIS TOPIC**

- Cancer vaccines can elicit antigen-specific immune responses that could be potentiated by the strategic combination of ICIs. However, they are often unsuccessful in inducing tumor eradication and sustained anti-tumor responses, failing to overcome tumor heterogeneity, immunosuppressive tumor microenvironments, and existing immune tolerance to tumor antigens (TAs). Photothermal therapy (PTT) is known to induce immunogenic cell death (ICD) of tumor cells and liberate TAs that the host immune system could specifically recognize, which may be potentiated by in situ immune modulators. High temperature accompanying PTT may be detrimental to the functionality of immune modulators administered in situ. Immune checkpoint inhibitors (ICIs) potentiate or reinvigorate pre-set anti-tumor immune responses.

**WHAT THIS STUDY ADDS**

- This study explores a novel strategy combining PTT employing tumor tissue targeting nanoparticle carrying thermoresistant flagellin, flagellin-adjuvanted cancer vaccine (FlaB-Vax), and anti-PD-1 ICI. The RGD-decorated targeted liposomal formulation (TLIF) encapsulating a photosensitizer and thermoresistant in situ adjuvant flagellin optimized PTT to induce robust ICD liberating immunogenic TAs resulting in epitope expansion. FlaB-Vax primed tumor-specific CD8^+^ T cells, which synergized with PTT-mediated antigen expansion and immune checkpoint inhibition, resulting in longer-lasting immune memory to prevent recurrence.

**HOW THIS STUDY MIGHT AFFECT RESEARCH, PRACTICE, OR POLICY**

- This study shows that the TLIF-PTT-released TAs, adjuvanted in situ with thermoresistant flagellin, synergize with FlaB-Vax-primed CD8^+^ T cells in inducing strong tumor-specific immune responses to suppress tumor recurrence, which could be further potentiated with ICIs. This provides new immunotherapeutic strategies against intractable cancers that have a propensity to recur and metastasize. The findings would provide a practical/scientific foundation for combining ICD-inducing tumor burden removal, host immune priming with vaccination, and potentiation of tumor-specific immune responses with ICIs.

## Introduction

 Given that cancers arise from mutations in normal cells and mutated proteins in cancer cells are recognized as foreign to immune cells, immunotherapeutic regimens have been tried for many decades. To immunologically combat cancer cells, many diverse modalities covering a broad spectrum of innate and adaptive immune compartments were applied to various tumors, which proved unsuccessful in saving lives in the majority of cases. The introduction of immune checkpoint inhibitors (ICIs) to cancer treatments drastically changed the cancer therapeutic landscape.^1^,^4^ However, accumulating therapeutic outcome data shows that only a limited fraction of cancer patients has been responsive to ICI therapies. This limited responsiveness is attributed to the complex immunosuppressive tumor microenvironment (TME), immune escape, tumor antigen (TA) heterogeneity, immune tolerance, etc.^5^ ICIs bring better therapeutic outcomes in combination with other modalities such as targeted therapeutics, immunogenic chemotherapeutics, other ICIs, radiotherapy, cancer vaccines, etc. Cancer vaccines have recently been highlighted thanks to the quantum developments in gene sequencing and computational tools.^6 7^

Cancer vaccines elicit TA-specific immune responses and establish long-term immune memory, aiming at eradicating cancer cells and suppressing recurrence/metastasis.^8^,^10^ However, they often reveal insufficiency in inducing tumor-eradicating and sustained tumor suppression.^11^ TA heterogeneity, immunosuppressive TMEs, and immune tolerance mechanisms pose significant challenges to cancer vaccines.^12^,^14^ Identifying suitable TAs, either tumor-associated antigens (TAAs) or tumor-specific antigens (TSAs), including mutated neoantigens and optimal adjuvants, is critical for successful cancer vaccine development, which should be variable among different types of cancers.^6 15^

Breast cancer is a high-mortality malignant disease affecting women worldwide. Historically, breast cancer was treated as a locoregional disease with surgery, which contributed to considerable life-saving throughout all age groups worldwide. However, metastatic breast cancer is the major cause of death in the aging female population over the age of 40, according to the The Surveillance, Epidemiology, and Results (SEER) database (https://seer.cancer.gov/statfacts/html/breast.html). The cancer cells can spread to several body parts, commonly to the lungs, bones, liver and brain. As cancer cells spread regionally or distantly, the 5-year mortality escalates exponentially, according to the SEER database. Immunotherapy against breast cancers should aim at the prevention and suppression of metastasis. In this context, we adopted the DD-Her2/neu cancer implantation model^16^ to pursue an efficacious way of achieving long-lived suppression of recurrence and metastasis after locoregional breast cancer removal.

To achieve efficacious eradication of large established tumors, combining multiple immunotherapeutic modalities engaging innate and adaptive immune responses was required to overcome immunosuppressive TME.^16^ They used quaternary combination immunotherapy: a TA targeting antibody to reduce tumor burden, an extended half-life recombinant half-life interleukin-2 for the expansion of effector lymphocytes, anti-PD-1 for boosting anti-tumor immune responses, and a potent T cell vaccine to trigger specific anti-tumor T cell responses.^16^ It was demonstrated that CD8^+^ T cells, cross-presenting Dendritic cells (DCs), and several other innate immune cell subsets were required for tumor regression. The tumor-eradicating combinatorial immunotherapy, accompanied by the infiltration of immune cells and the production of inflammatory cytokines in the tumor, promoted antigen spreading. Considering these findings, in the present study, we have designed a tripartite immunotherapeutic strategy that would achieve efficacious tumor eradication and sustained suppression of recurrence/metastasis: photothermal therapy aiming tumor burden lessening and immunogenic tumor cell death, thermoresistant TLR5 ligand-adjuvanted TAA vaccine to trigger tumor-specific immune responses and anti-PD-1 for invigorating induced anti-tumor immune responses.

Phototherapy, including photodynamic therapy (PDT) and photothermal therapy (PTT), has shown significant potential in cancer treatment.^17^ PTT uses near-infrared (NIR) light to generate localized heat, leading to precise tumor cell ablation with minimal side effects.^18 19^ Indocyanine green (ICG), a clinically approved NIR dye, is widely used in PTT.^20 21^ However, PTT-induced immunogenic cell death (ICD) might not be enough to stimulate long-lasting immune responses.^22^ PTT inevitably generates local heat that negatively affects therapeutic agents reached at tumor sites. Biomolecules would be labile to PTT-generated local heat, limiting the effectiveness. Flagellin serves as a potent adjuvant to diverse types of vaccines by activating dual signaling pathways, specifically TLR5 and NLRC4.^23^,^25^ Its heat stability makes it an ideal combinatorial partner for PTT.^26^

In this study, we hypothesize that combining PTT with a flagellin-adjuvanted cancer vaccine (FlaB-Vax) will bring a synergistic effect in the DD-Her2/neu mouse breast cancer model. To test this end, we developed a targeted liposomal formulation (TLIF) encapsulating ICG and flagellin to maximize PTT effects. Here, encapsulated flagellin will serve as an in situ adjuvant for ICD-generated TAs. Using the DD-Her2/neu tumor model, we investigated the antigen-specific immune responses induced by TLIF-mediated PTT and FlaB-Vax. To trigger a TAA-specific CD8^+^ cytotoxic T lymphocyte (CTL) response that will prime the host immune system, we formulated flagellin with DD-Her2/neu-specific CTL peptide.^16^

## Materials and methods

### Synthesis and characterization of Cyclic RGD (cRGD) liposomes encapsulating indocyanine green (ICG) and flagellin (FlaB) (cRGD-lipo-ICG-FlaB); targeted liposomal formulation (TLIF) nanoparticle

**Figure 1:**
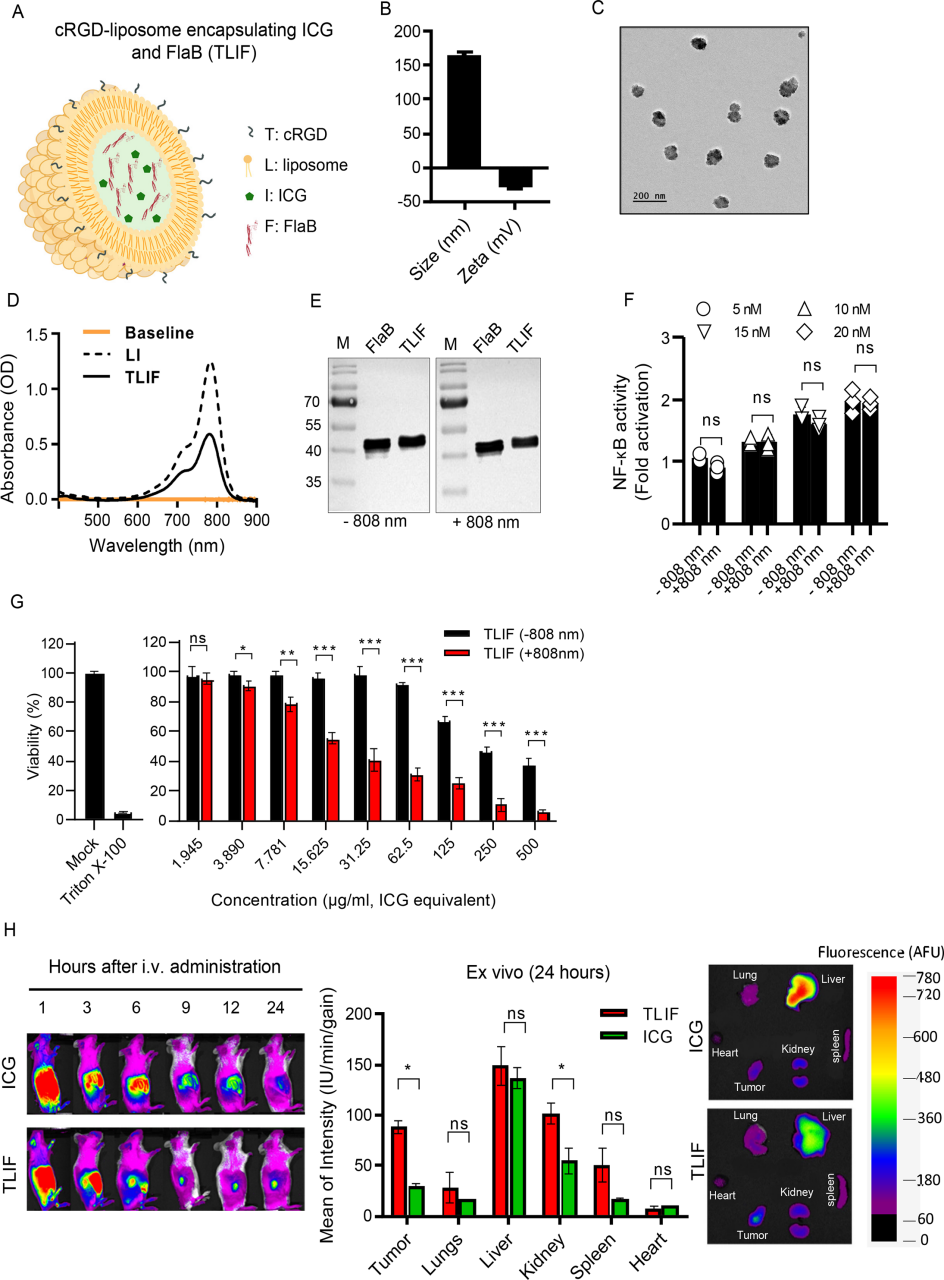
Preparation of cRGD-liposome encapsulating indocyanine green (ICG) and flagellin (targeted liposomal formulation (TLIF)) for photothermal therapy (PTT) and determination of tumor tissue targeting. (A) Schematic representation of the preparation of encapsulated photosensitizer ICG and immune modulator flagellin (FlaB). (B) Hydrodynamic size and zeta potential of TLIF determined by dynamic light scattering (DLS). (C) Representative field emission transmission electron microscopy (FE-TEM) images of liposome (TLIF). (D) Ultraviolet visible near-infrared (NIR) absorption spectra of TLIF and liposome encapsulating ICG (LI). (E) Western blot analysis of FlaB and TLIF with and without 808 nm laser treatment at 2 W/cm^2^ for 5 min. The analysis was performed to assess the stability of FlaB and TLIF after in vitro PTT. The control lanes represent untreated samples, while the treated lanes represent samples exposed to the 808 nm laser. (F) Determination of TLR5-stimulation activity by TLIF following 808 nm laser irradiation at 2 W/cm² for 5 min. The experiment evaluated the functional activity of released FlaB from TLIF in stimulating TLR5 after PTT. (G) Viability of DD-Her2/neu cells after co-incubation with TLIF nanoparticles and subsequent treatment with 808 nm laser irradiation at 2 W/cm² for 5 min of PTT. The analysis was conducted to assess the effect of TLIF nanoparticles and PTT on cell viability. (H) Biodistribution of TLIF after intravenous administration. Representative ex vivo fluorescence imaging and fluorescence intensity analysis of major organs and tumors, showing the distribution of TLIF at 24 hours post-injection (n=3). Data are presented as the mean±SEM. Statistical significance was determined using an unpaired t-test or two-way analysis of variance and Tukey’s multiple comparison test. Statistical significance is defined as follows: ns p>0.05 (ns indicates non-significance), *p<0.05, **p<0.01, ***p<0.001.

The cRGD liposomes encapsulating ICG and FlaB (TLIF, figure 1A) were synthesized using the thin-film hydration method. Initially, cRGD liposomes were prepared as previously described.^27^ The stability and presence of FlaB in TLIF were confirmed by Sodium Dodecyl Sulfate-Polyacrylamide Gel Electrophoresis (SDS-PAGE) analysis followed by Western blotting, using an anti-FlaB antibody.^28^,^30^ Additional information related to nanoparticle preparation and released FlaB immune activation assay can be found in online supplemental information.

### Peptide and recombinant protein

The synthetic peptide corresponding to amino acids 66–74 of the Her2/neu protein (p66, CTYVPANASL) was used as a peptide vaccine antigen.^16 31 32^ It was synthesized by AnyGen Co., Ltd. (Gwangju, South Korea). *V. vulnificus* flagellin B (FlaB) was used as an adjuvant and active component of the liposome (cRGD-lipo-ICG-FlaB; TLIF) nanoparticle. The recombinant FlaB was prepared as previously described.^24 33^

### Cell culture

Glenn Dranoff from the Dana-Farber Cancer Center generously provided the DD-Her2/neu cell line.^15^ These cells were cultured in RPMI 1640 medium (Life Technologies, Grand Island, NY, USA) supplemented with 10% fetal bovine serum (HyClone, Logan, UT) and 100 units/ml penicillin and 100 µg/mL streptomycin (Life Technologies, Grand Island, NY, USA). Cultures were maintained at 37°C in a humidified atmosphere with 5% CO_2_.

### Determination of cytotoxic effects

The cytotoxic effects of TLIF were assessed in pre- and post-laser irradiation conditions using the DD-Her2/neu cell line. The online supplemental information provides additional information related to the cytotoxicity assay.

### Drug release study

Detailed experimental information is provided in Online supplemental methods.

### Photostability of cRGD liposomes encapsulating indocyanine green (ICG) and flagellin (FlaB) (cRGD-lipo-ICG-FlaB); targeted liposomal formulation (TLIF) nanoparticle

Detailed experimental information is provided in supplementary methods.

### Investigation of indocyanine green (ICD) evaluation in vitro

Detailed experimental information is provided in the supplementary methods.

### Biodistribution assessment of targeted liposomal formulation (TLIF)

Female Balb/c mice, aged 6–8 weeks, were used to investigate the in vivo TLIF biodistribution. The signal intensities were quantitatively analyzed by measuring the maximum photons per second per square centimeter per steradian (p/s/cm²/sr). The online supplemental information provides details of the biodistribution assay.

### Optimization of photothermal therapy (targeted liposomal formulation (TLIF)-photothermal therapy (PTT))

To determine the optimal PTT conditions that provide strong immune responses, we irradiated TLIF-accumulated tumors at different temperatures. The online supplementary methods provide detailed information.

### Combination of targeted liposomal formulation (TLIF)-photothermal therapy (PTT) and flagellin (FlaB)-adjuvanted peptide cancer vaccine (TLIF-PTT+FlaB-Vax) in bilateral DD-Her2/neu mice model

In the bilateral tumor experimental setup, the primary tumor was established by subcutaneous injection of 5×10^6^ DD-Her2/neu cells in 100 µL of PBS into the right flanks of female Balb/c mice (figure 3A). Subsequently, the secondary tumor was induced by injecting 1×10^6^ DD-Her2/neu cells in 100 µL of PBS into the left flanks the next day. Once the primary tumors reached a diameter of 3–5 mm, the mice were randomly divided into several treatment groups: a non-treated group (Control (Con)), a group receiving the FlaB-adjuvanted peptide vaccine (FlaB-Vax(V)), a group for PTT (TLIF-PTT(P)), and a group receiving a combination of PTT and the vaccine (TLIF-PTT+FlaB Vax (PV)). In the FlaB-Vax and TLIF-PTT+FlaB Vax groups, the vaccine was administered peritumorally. Each dose of the vaccine comprised 4 µg of FlaB and 100 µg of the Her2 peptide (p66; TYVPANASL). Vaccination was performed 3 days before the start of TLIF-PTT and continued on days +3 and +6 after PTT. The TLIF-PTT and TLIF-PTT+FlaB Vax groups received intravenous injections of lipoICG (LI) or cRGD-lipoICG-FlaB (TLIF) formulation at an equivalent dose of 5 mg/kg ICG 1 day before the laser irradiation. The PTT and TLIF-PTT+FlaB Vax groups then underwent light irradiation at 808 nm and a power density of 2 W/cm², maintained at 50°C for 5 min, 24 hours postinjection of nanoparticles. Tumor volume was monitored at 3 day- intervals using the formula: *V = (tumor length×tumor width×tumor height*) / 2. Ethical euthanasia was performed on reaching the tumor volume of 2000 mm³ in the primary or secondary tumor site.

### Triple combination of targeted liposomal formulation (TLIF)-photothermal therapy (PTT), flagellin-adjuvanted cancer vaccine (FlaB-Vax), and αPD-1 in an orthotopic DD-Her2/neu breast cancer model

We performed a triple combination of TLIF-PTT, FlaB-Vax, and αPD-1 in an orthotopic DD-Her2/neu breast cancer model. The supplementary methods provide detailed information.

### Rechallenge in survivors of the triple combination therapy

Mice from the TLIF-PTT+FlaB-Vax+αPD-1 (PVI) group that achieved complete tumor eradication until 250 days after treatment were selected for the rechallenge experiment. Tumor eradicated mice and age-matched naive mice underwent rechallenge by implanting 1×10^6^ DD-Her2/neu cells into the colorectal region fat pad (left mammary gland). Tumor volume was monitored and calculated using the formula: *V = (tumor length×tumor width×tumor height) /* 2.

### Detection of Her2-specific CD8^+^ T cells

Detailed experimental information is provided in the supplementary methods.

### Detection of Her2-specific interferon gamma (IFN-γ) production using Enzyme-Linked Immunospot (ELISpot) analysis

Detailed experimental information is provided in the supplementary methods.

### Detection of therapy-mediated antibody responses in the blood

To detect DD-Her2/neu-specific antibody responses following therapy, Western blot analysis was performed using sera collected from the treated mice. Detailed information is provided in the supplementary methods.

### Detection of lung metastasis in orthotopic DD-Her2/neu mice

On day 7 post-treatment, mice were euthanized to assess lung metastasis.^23^ India ink was intratracheally injected to facilitate visualization. Lungs were extracted and immersed in Fekete’s solution for destaining (100 mL 70% ethanol, 10 mL 4% formaldehyde, and 5 mL 100% glacial acetic acid). Metastatic nodules were counted with the naked eye.

### Ethics statement

All experimental animal procedures were approved by the Chonnam National University Institutional Animal Care and Use Committee under protocol CNU IACUC-H-2023–48. Animal research facility maintenance and experimental procedures adhered strictly to the guidelines in the Animal Welfare Act enacted by the Korean Ministry of Agriculture, Food, and Rural Affairs.

### Statistical analysis

Data analysis was conducted using GraphPad Prism 9. Results are expressed as mean±SEM unless otherwise specified. Statistical significance was assessed using one-way analysis of variancce (ANOVA) followed by Tukey’s multiple comparisons test or two-way ANOVA with Bonferroni post hoc analysis. Survival analysis was performed using the Kaplan-Meier method. The log-rank Mantel-Cox test was employed to compare the survival distributions between two groups. Statistical significance was defined as follows: ^ns^ p=0.05 or higher (ns indicates non-significance), *p<0.05, **p<0.01, and ***p<0.001.

## Results

### Preparation of cRGD-liposome encapsulating indocyanine green (ICG) and flagellin for photothermal therapy (PTT) and determination of tumor targeting

A cRGD-displaying liposome^27^ loaded with ICG and flagellin (FlaB) was synthesized using the lipid film hydration method followed by extrusion.^34^ This nanoparticle formulation, named TLIF (figure 1A), demonstrated good water solubility with a hydrodynamic diameter of 155±15 nm and a zeta potential of approximately −20 mV when successfully encapsulated both ICG and FlaB (figure 1B). Transmission electron microscopy (TEM) images revealed a uniform granular morphology with a diameter of ~100 nm, which swelled to the average hydrodynamic diameter in aqueous solutions (figure 1C). The characteristic absorption peak at 771 nm confirmed successful ICG loading in TLIF in (figure 1D). The loading efficiency of ICG and FlaB was calculated using the following formula: Loading efficiency (%) = (weight of cargo / weight of nanoparticle) × 100. The loading efficiency of ICG in cRGD-lipo-ICG-FlaB (TLIF) was 16.42±4.07% while that of FlaB was 76.32±4.00%. These results indicate a compromise in loading efficiency when encapsulating both FlaB and ICG together. Additionally, the encapsulation efficiency (EE) of ICG and FlaB was calculated using the following formula: Encapsulation efficiency (%) = (weight of cargo / weight of cargo feed) × 100. The EE of ICG was 56.1±3.3% and that of FlaB was 58.2±3.6%. Furthermore, as shown in online supplemental figure S1a, the release profile of ICG from TLIF was monitored using ultraviolet-visible NIR spectrophotometry in 500 µl of PBS containing 0.2% Tween 80. Non-encapsulated free ICG exhibited rapid release, while TLIF demonstrated a controlled and sustained release of ICG. This result indicates that TLIF effectively encapsulates ICG, slowing its release and potentially extending its functional duration in therapeutic situations. The cumulative release profile of FlaB from TLIF is presented in online supplemental figure S1b. Non-encapsulated free FlaB displayed a faster release rate: complete release was observed in 6 hours. In comparison, FlaB appeared to be well contained in TLIF: over 85% of loaded FlaB remained in TLIF even after 48 hours. These findings confirm that the TLIF nanoparticle system provides a stable encapsulation environment for FlaB. The controlled and sustained release profiles of both ICG and FlaB from TLIF highlight its usefulness as a multifunctional nanoparticle system for combined photothermal and immunotherapeutic applications. As shown in online supplemental figure S2a–c), the temperature change profiles of TLIF nanoparticles were evaluated at different ICG concentrations (5, 10, 20, and 40 µg/mL) under 808 nm laser irradiation at 2 W/cm² for 5 min. The temperature increase appeared to be dependent on TLIF concentration, where 5 µg/mL was relatively weaker in increasing temperature, while 10 µg/mL or higher concentration showed a similar temperature rising pattern. To assess the stability of the nanomedicine under repetitive NIR exposures, we performed a photothermal cycling test at 10 µg/mL using 2 W/cm² power density (online supplemental figure S2c). The uncompromised temperature elevation patterns over repeated NIR exposure suggested excellent photothermal stability of cRGD-Lipo-ICG-FlaB (TLIF), which would serve as a requisite for clinical applications. Western blot analysis (figure 1E) shows that the integrity of FlaB is well conserved after the 808 nm irradiation in both free and TLIF incorporated states. The TLR5-activating functionality was also unharmed by the PTT (figure 1F). The dose-dependency of TLR5 activation was also well conserved after the NIR irradiation of TLIF nanoparticles. These results indicate that TLIF will effectively activate the immune system under various PTT conditions.

To see whether TLIF-PTT kills cancer cells, DD-Her2/neu cells were incubated with TLIF for 6 hours and irradiated with 808 nm NIR light at 2 W/cm² for 5 min. Then, cell viability was determined using the water-soluble tetrazolium salt assay. As shown in figure 1G, the viability of irradiated DD-Her2/neu cells significantly decreased across a wide range of TLIF concentrations (3.890 to 500 µg/mL). PTT is known to be an excellent inducer of ICD.^35^,^39^ We tested whether TLIF-PTT could significantly induce ICD. The ICD-inducing ability of TLIF was compared with ICG using an in vitro DD Her2/neu culture system. After incubating the cells in the presence of TLIF or ICG for 4 hours, NIR irradiation was carried out, and the expression of ICD hallmarks, calreticulin (CRT) and high mobility group box 1 (HMGB1) protein, was assessed. Without NIR irradiation, both TLIF and ICG did not induce any discernable ICD by 5 min. 2 W/cm^2^ 808 nm laser irradiation. ICG could induce obviously detectable levels of CRT and HMGB1 after the NIR irradiation. On the other hand, TLIF-incubated DD Her2/neu cells underwent significantly enhanced ICD (online supplemental figure S3). Typically, CRT was associated with cell surface (online supplemental figure S3a), and HMGB1 was mobilized to nuclei (figure 3B), which attests to the typical feature of ICD. CRT displayed on the surface of ICD-undergoing cells serves as a Damage-Associated Molecular Pattern (DAMP) stimulating innate immune cells such as DCs. HMGB1 released from cells committed to ICD is also a strong DAMP enhancing immune responses. ICD of tumor cells by the TLIF-PTT should has provided TAs for epitope expansion as well as DAMP signals cooperating with the flagellin-TLR5 signaling axis.

Encouraged by this significant photothermal killing efficacy in vitro, we subsequently examined TLIF’s tumor tissue accumulation ability using DD-Her2/neu-tumor-bearing mice. When the tumor volume reached approximately 5–8 mm in diameter, the mice were randomly divided into two groups and administered with ICG or TLIF. TLIF appeared preferentially confined in the tumor tissue compared with ICG, which became more evident after 24 hours (figure 1H), highlighting TLIF’s tumor-targeting ability. The ex vivo quantitative imaging further corroborated these findings, showing significantly enhanced accumulation of TLIF in the tumor, suggesting the enhanced permeability and retention (EPR) effect in tumor tissue and cRGD-mediated active retention. A significant accumulation of TLIF was noted in the liver, kidney, and spleen, where the nanoparticles were supposed to be cleared (figure 1H).

### Optimization of targeted liposomal formulation (TLIF)-photothermal therapy (PTT) in a DD-Her2/neu orthotopic breast cancer model

**Figure 2:**
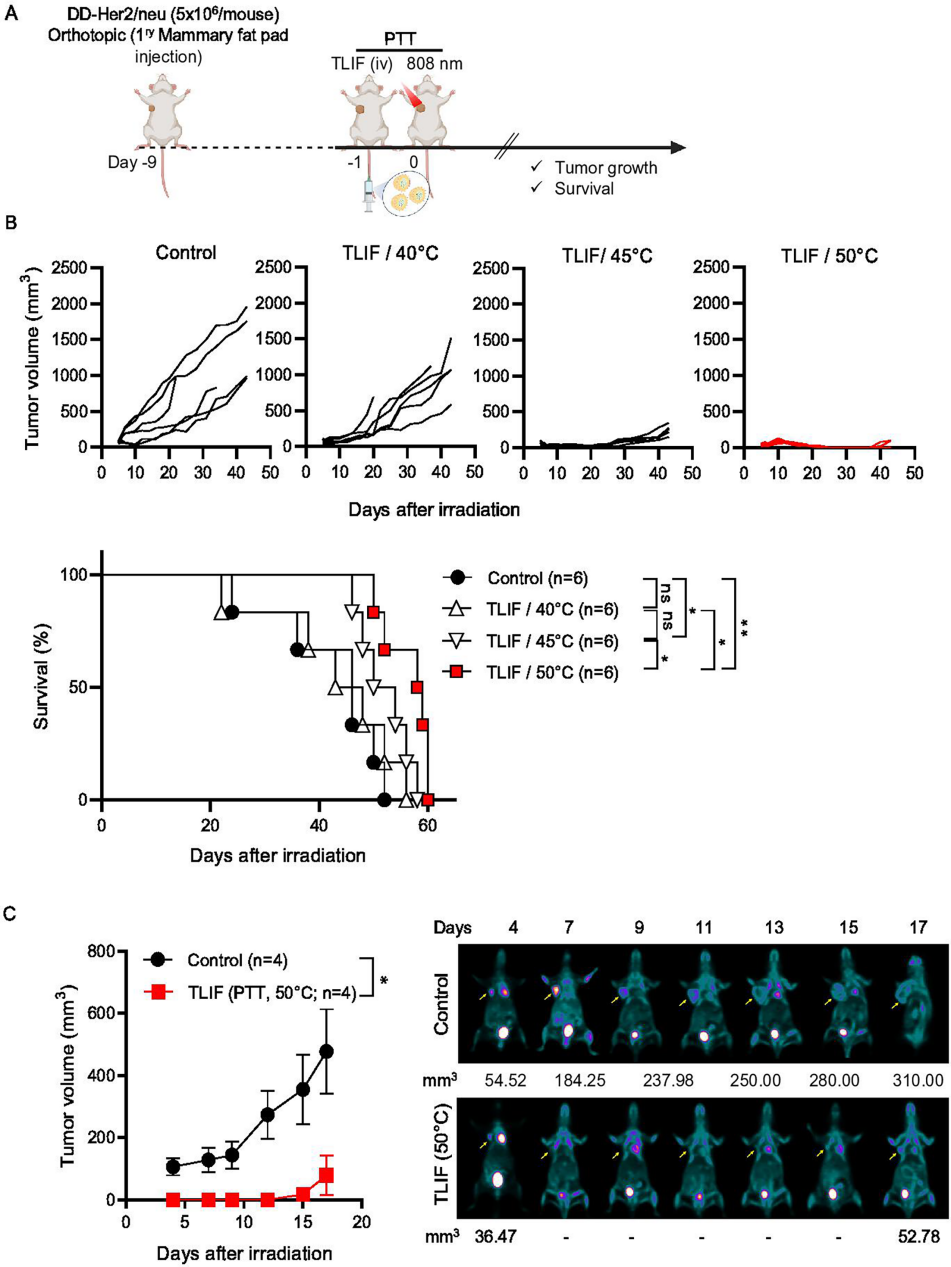
Optimization of photothermal therapy (PTT) conditions using targeted liposomal formulation (TLIF) in a DD-Her2/neu breast orthotopic mouse model. (A) Schematic of the TLIF-induced PTT (TLIF-PTT) schedule in the DD-Her2/neu breast orthotopic mouse model. (B) Tumor growth curves of DD-Her2/neu tumor-bearing mice after different PTT temperature settings and survival proportion (n=6). The log-rank (Mantel-Cox) test was employed to compare the survival distributions between the two groups. (C) Tumor growth curves based on MicroPET imaging of DD-Her2/neu tumor-bearing mice treated with TLIF-PTT at 50°C. Data are presented as the mean±SEM. Statistical significance was determined using an unpaired t-test or the log-rank (Mantel-Cox) test. ns p>0.05 (ns, non-significance), *p<0.05, **p<0.01.

Having confirmed the preferential tumor accumulation ability of TLIF, we optimized the TLIF-PTT protocol using a DD-Her2/neu orthotopic breast cancer model by varying the tumor temperature elevated by the 808 nm NIR irradiation (figure 2A). We wanted to know the optimal ICG activation-mediated temperature elevation in the tumor tissue, inducing optimal ICD and robust antitumor immune responses. When the tumor reached approximately 5–8 mm in diameter, mice were randomly divided into four groups for different treatments: (I) Control, (II) TLIF/40°C, (III) TLIF/45°C, and (IV) TLIF/50°C. All groups received 808 nm NIR laser irradiation 24 h post-TLIF. As shown in figure 2B, the TLIF/40°C treatment did not show statistically significant tumor suppression. The TLIF/50°C group demonstrated a significant delay in tumor growth and improved survival, whereas the TLIF/45°C group showed recognizable tumor growth but shorter survival. This result suggested that the tumor tissue temperature elevation will affect ICD and subsequent anti-tumor immune responses. The significant tumor-suppressive efficacy of TLIF/50°C PTT was also clearly demonstrated by the microPET imaging (figure 2C).

### Long-term survival of bilateral tumor-bearing mice treated with the combination of targeted liposomal formulation (TLIF)-photothermal therapy (PTT) and flagellin-adjuvanted cancer vaccine (FlaB-Vax)

**Figure 3:**
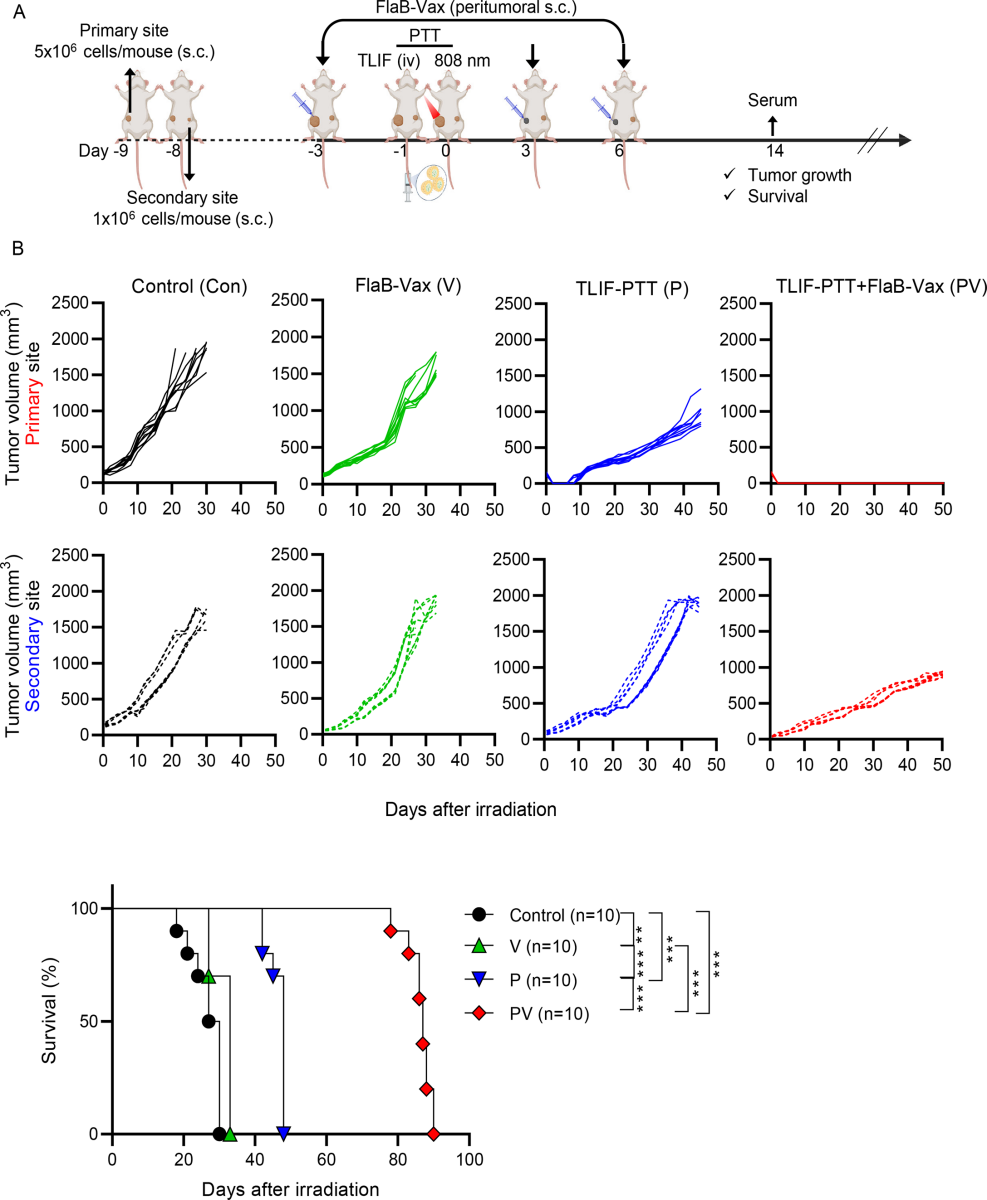
Long-term survival of bilateral tumor-bearing mice treated with the combination of targeted liposomal formulation (TLIF)-photothermal therapy (PTT) and flagellin-adjuvanted cancer vaccine (FlaB-Vax). (A) Schematic representation of the TLIF-PTT and FlaB-Vax combination therapy schedule in a DD-Her2/neu bilateral tumor implantation model. (B) Growth kinetics of irradiated primary and non-irradiated secondary tumors (n=10) and survival proportion of mice treated with TLIF-PTT, FlaB-Vax, or TLIF-PTT+FlaB Vax (n=10). The log-rank (Mantel-Cox) test was employed to compare the survival distributions between the two groups. Statistical significance is defined as follows: ns p>0.05 (ns, non-significance), *p<0.05, **p<0.01, ***p<0.001.

Flagellin, a TLIF component, is an efficacious adjuvant for therapeutic cancer vaccines^23^,^2534^ and has immune-modulating activity in the TME.^32 40^ Flagellin induces TLR5 expression of dLN DCs in a positive-feedback fashion.^24 33^ We hypothesized that the TLIF-PTT and peritumoral FlaB-Vax would synergize in this context. PTT would liberate FlaB from the nanoparticles accumulated in tumors, which will drain into adjacent LNs, along with TAs released from cancer cells undergoing ICD. The first peritumoral vaccination would prime dLN DCs that become sensitized to be more responsive to the FlaB and TAs released by PTT (figure 3A). In addition, to assess the combination therapy-induced systemic immune responses, we evaluated the abscopal tumor suppression using a bilateral tumor implantation model (figure 3A). When the tumor diameter at the primary site reached 5–8 mm, the mice were irradiated with 808 nm NIR at the primary site. The day of NIR irradiation was designated as day 0. For the FlaB-Vax group, tumor-bearing mice were immunized with 100 µg of TYVPANASL peptide and 4 µg of FlaB three times on days −3, 3, and 6. As shown in figure 3B, in the combination group, the primary tumor growth was completely suppressed, and the growths of secondary tumors were also significantly retarded compared with other experimental groups. This result demonstrates that TLIF-PTT and FlaB-Vax were synergistic in the suppression of primary and abscopal tumors. FlaB-Vax showed no tumor suppression compared with TLIF-PTT in both primary and abscopal tumors. Additionally, both FlaB-Vax and TLIF-PTT significantly extended survival. Notably, the combination of TLIF-PTT with FlaB-Vax dramatically extended the survival compared with monotherapies (p<0.001). This result suggests that the specific immune response primed with the Her2 peptide antigen synergized with the immune responses against TAs released by the PTT.

### Potentiation of tumor-specific cellular immune responses by the combination of targeted liposomal formulation (TLIF)-photothermal therapy (PTT) and flagellin-adjuvanted peptide vaccine (FlaB-Vax)

**Figure 4:**
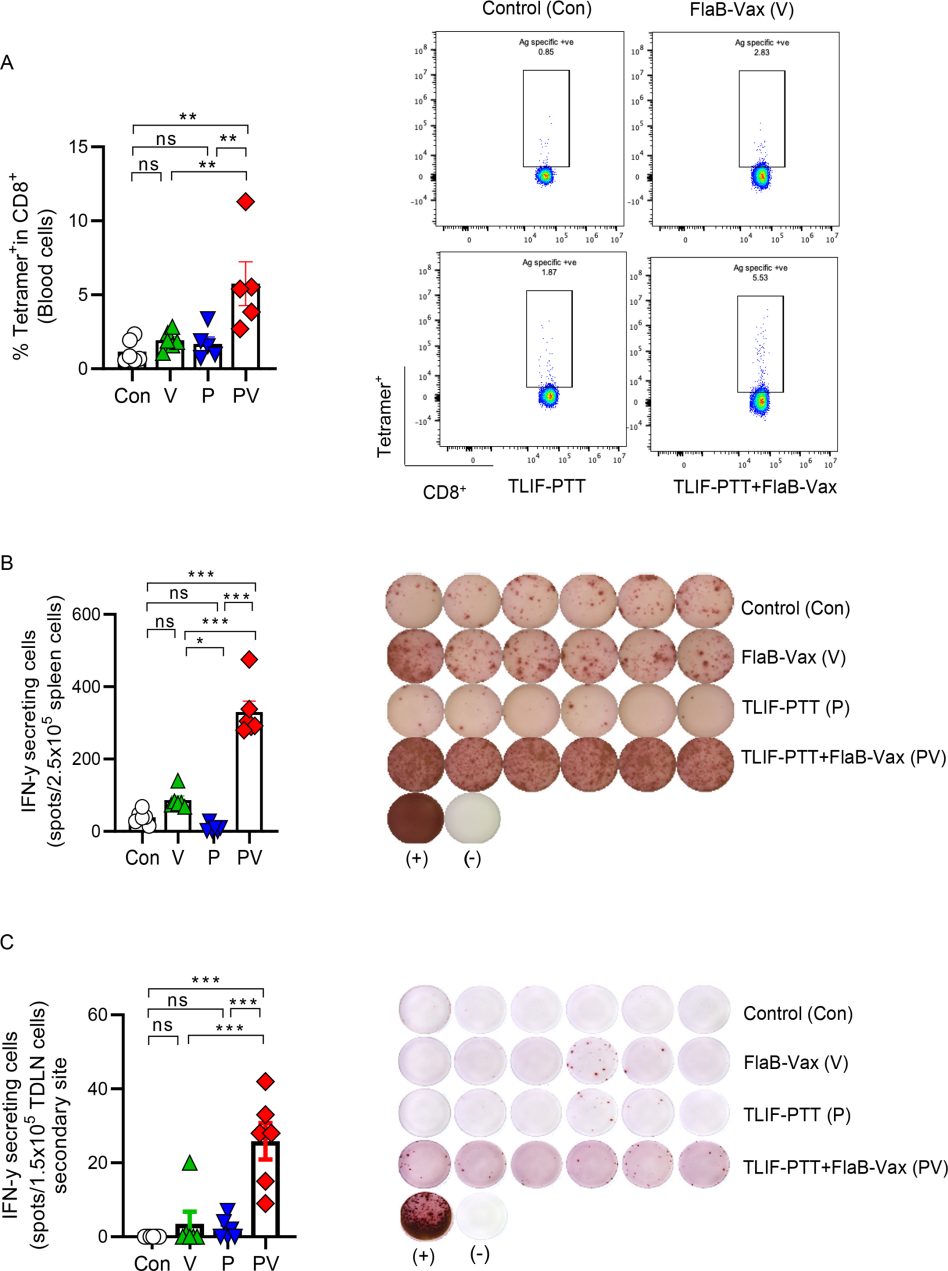
Potentiation of tumor-specific cellular immune responses by the combination of targeted liposomal formulation (TLIF)-photothermal therapy (PTT) and flagellin-adjuvanted peptide vaccine (FlaB-Vax). (A) Determination of Her2 CTL epitope (p66; TYVPANASL)-specific tetramer-positive cells in peripheral blood CD8^+^ cells assessed by flow cytometry (n=5 or 6). (B) ELISpot analysis of Her2 CTL epitope (p66; TYVPANASL)-specific interferon gamma (IFN-γ)-producing cells in 2.5×10^5^ spleen cells (n=6). (C) ELISpot analysis of IFN-γ-secreting cells in tumor-draining lymph node (TDLN) cells isolated from secondary sites on day 7 after final treatment. Cells were stimulated with Her2 peptide (n=6, 1.5×10^5^ cells per well). Data are presented as the mean±SEM. Statistical significance was determined using an ordinary one-way analysis of variance and Tukey’s multiple comparison test. Statistical significance is defined as follows: ns p>0.05 (ns, non-significance), *p<0.05, **p<0.01, ***p<0.001. Con, Control; V, FlaB-Vax; P, TLIF-PTT; PV, TLIF-PTT+FlaB Vax.

To assess the underlying mechanisms responsible for the synergistic tumor suppression and prolonged survival by the TLIF-PTT and FlaB-Vax combination therapy, we measured antigen-specific immune responses in the blood, spleen, and tumor-draining lymph nodes (tdLNs) on day 14. At this point, there were no observed differences in distant tumor size between groups. Since primary tumors were completely eradicated in mice treated by TLIF-PTT and FlaB-Vax combination, we analyzed immune cells in the tdLNs of abscopal sites. To detect TA-specific CD8^+^ cells in the systemic compartment, we measured Her2 (p66; TYVPANASL) peptide-specific tetramer-positive CD8^+^ cells in the blood. As shown in figure 4A, the TLIF-PTT+FlaB Vax group exhibited a significantly higher number of antigen-specific CD8^+^ T cells (***p<0.01 for Control vs TLIF-PTT+FlaB Vax; *p<0.1 for FlaB-Vax vs PTT+FlaB Vax; **p<0.01 for PTT vs PTT+FlaB Vax). In addition, the TLIF-PTT+FlaB Vax group induced significantly higher numbers of Her2 (p66; TYVPANASL) peptide-specific interferon gamma (IFN-γ)-producing cells in the spleen (***p<0.001 for Control vs TLIF-PTT+FlaB Vax; ***p<0.001 for FlaB-Vax vs TLIF-PTT+FlaB Vax; ***p<0.001 for TLIF-PTT vs TLIF-PTT+FlaB Vax) (figure 4B). Of note, the FlaB-Vax group exhibited a significantly higher number of peptide-specific IFN-γ producing splenocytes than the TLIF-PTT group, suggesting that the vaccination induced significant peptide antigen-specific immune responses (figure 4B). These results confirm that the TLIF-PTT enhanced the peptide antigen-specific cellular immune responses induced by FlaB-Vax immunization. To ascertain whether the PTT-mediated tumor irradiation at the primary site may amplify systemic immune responses that would affect abscopal tumors, we determined Her2 (p66; TYVPANASL) peptide-specific IFN-γ producing cells in the tdLNs of abscopal tumors. Notably, significantly higher numbers of Her2-specific IFN-γ producing cells were detected in the TLIF-PTT+FlaB Vax group compared with monotherapy groups (***p<0.001 for FlaB-Vax vs TLIF-PTT+FlaB Vax; ***p<0.001 for TLIF-PTT vs TLIF-PTT+FlaB Vax) (figure 4C). These results strongly testify that the systemically induced specific immune responses by combining TLIF-PTT with FlaB-Vax were responsible for the abscopal tumor suppression.

### Inhibition of tumor spreading by the combination of targeted liposomal formulation (TLIF)-photothermal therapy (PTT) and flagellin-adjuvanted peptide vaccine (FlaB-Vax) in an orthotopic breast cancer model

**Figure 5:**
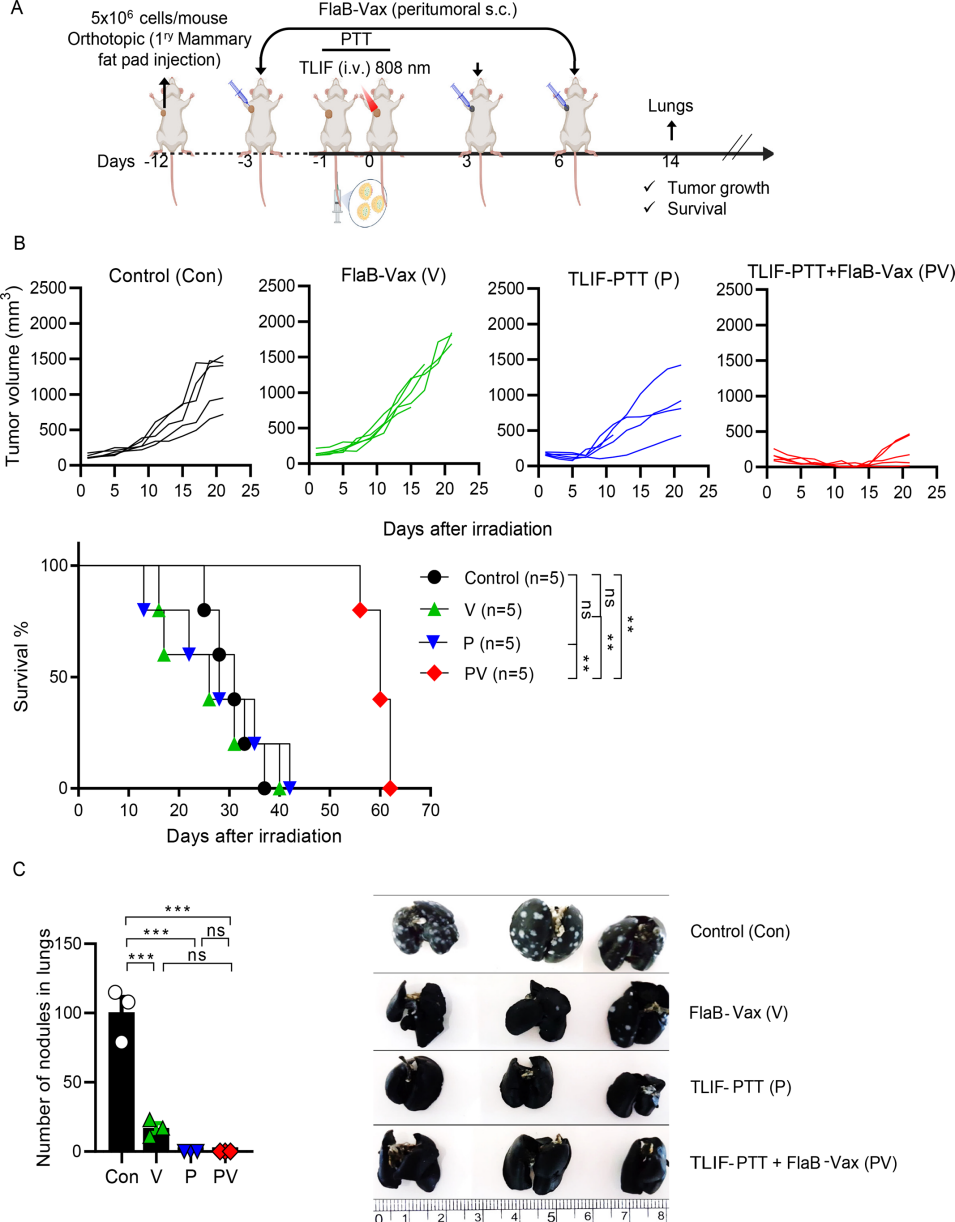
Inhibition of tumor spreading by the combination of targeted liposomal formulation (TLIF)-photothermal therapy (PTT) and flagellin-adjuvanted peptide vaccine (FlaB-Vax) in an orthotopic breast cancer model. (A) Schematic representation of the TLIF-PTT and FlaB-Vax combination therapy schedule in a large established DD-Her2/neu breast orthotopic mouse model. (B) Tumor growth kinetics in a large established DD-Her2/neu breast orthotopic mice model (n=5) and survival proportion of mice treated with TLIF-PTT, FlaB-Vax, or TLIF-PTT+FlaB Vax (n=5). The log-rank (Mantel-Cox) test was employed to compare the survival distributions between the two groups. (C) Determination of tumor spreading in the lung of orthotopic tumor-bearing mice on day seven post-treatment (n=3). Data are presented as the mean±SEM. Statistical significance was determined using an unpaired t-test or the log-rank (Mantel-Cox) test. Statistical significance is defined as follows: ns p>0.05 (ns, non-significance), *p<0.05, **p<0.01, ***p<0.001. Con, Control; V, FlaB-Vax; P, TLIF-PTT; PV, TLIF-PTT+FlaB-Vax.

Next, we investigated the therapeutic efficacy on tumor recurrence and metastasis in a large, established orthotopic tumor model. Based on the promising tumor-suppressive results of the combination therapy in the bilateral flank tumor model, we extended to a large established orthotopic breast cancer model. In large established tumors, heat distribution would not be homogeneous, and complete eradication of cancer cells would also be challenging. Large-established orthotopic breast tumors would have more chance to metastasize, which will allow observing how much the therapeutic strategy would be efficacious against distant metastasis. We established an orthotopic tumor in the mammary fat pad up to the volume of 150–200 mm³ (figure 5A). These large established orthotopic tumors would more closely simulate human breast cancer. In this model, although mice treated with TLIF-PTT+FlaB Vax initially showed tumor eradication, recurrence was observed 15 days after irradiation (figure 5B), indicating limited anti-tumor efficacy against large established tumors due to suboptimal heat dissipation in the tumor tissue. Pulmonary metastasis was examined on day 14 (figure 5A). The combination group manifested dramatically extended survival compared with other groups. The TLIF-PTT group’s survival did not differ from that of the FlaB-Vax or the control group, suggesting incomplete eradication of cancer cells by the treatment. Compared with non-treated tumor-bearing mice, all treated mice exhibited a significant reduction in lung metastatic nodules at the observed time point (figure 5C). Interestingly, no notable nodule was observed in the mice cured with TLIF-PTT and TLIF-PTT + FlaB Vax treatments on day 14, whereas the FlaB-Vax group still had 17±2.83 nodules. These results indicate that the combination of TLIF-PTT+FlaB Vax is efficacious in suppressing recurrence and metastasis on the orthotopic large established tumors.

### Induction of long-term relapse-free survival by a triple combination of targeted liposomal formulation (TLIF)-photothermal therapy (PTT), flagellin-adjuvanted peptide vaccine (FlaB-Vax), and anti-PD-1

**Figure 6:**
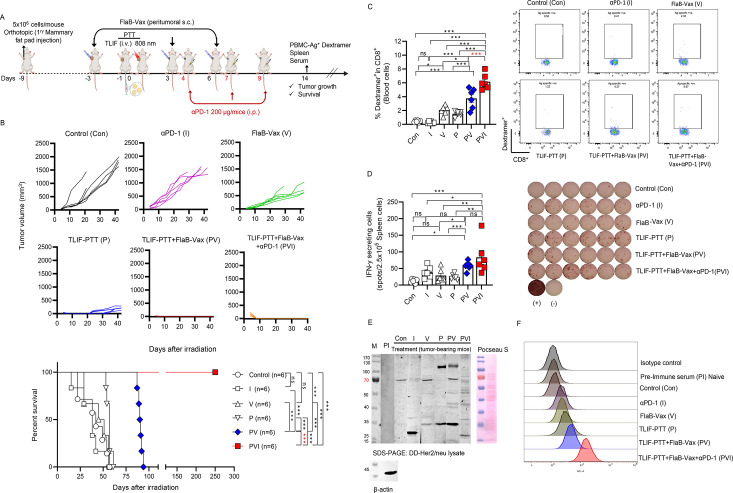
A triple combination of targeted liposomal formulation (TLIF)-photothermal therapy (PTT), flagellin-adjuvanted peptide vaccine (FlaB-Vax), and anti-PD1 induced long-term relapse-free survival, enhanced antigen-specific cellular immunity, and robust epitope expansion. (A) Schematic representation of the TLIF-PTT and FlaB-Vax combination therapy schedule in a DD-Her2/neu breast orthotopic mouse model. Vaccinations were administered on days −3, 3, and 6. Concurrently, αPD-1 or isotype control antibodies (200 µg/injection) were administered intraperitoneally (i.p.) on days 4, 7, and 9. (B) Tumor growth kinetics in a DD-Her2/neu breast orthotopic mice model (n=6) and survival proportion of mice treated with anti-PD1, FlaB-Vax, TLIF-PTT, TLIF-PTT+FlaB Vax, or TLIF-PTT+FlaB-Vax+ anti-PD1 (n=6). The log-rank (Mantel-Cox) test was employed to compare the survival distributions between the two groups. (C) Determination of Her2 CTL epitope (p66; TYVPANASL)-specific dextramer-positive cells in peripheral blood CD8^+^ cells assessed by flow cytometry (n=6). (D) ELISpot analysis of interferon gamma (IFN-γ)-secreting cells in spleen cells stimulated with Her2 peptide (n=6, 2.5×10^5^ cells per well). (E) Western blot analysis of DD-Her2/neu tumor cell lysate. Serum collected on day 14 after PTT was used to detect the antibody response, which was revealed with a mouse-IgG-specific secondary antibody. β-actin was used as a loading control. (F) DD-Her2/neu cells were incubated with 5% serum collected on day 14 after PTT. The cells were washed, stained with an APC-conjugated mouse-IgG-specific secondary antibody, and analyzed by flow cytometry. Statistical significance was determined using an unpaired t-test, log-rank (Mantel-Cox) test, ordinary one-way analysis, and Tukey’s multiple comparison test. Statistical significance is defined as follows: ns p>0.05 (ns, non-significance), *p<0.05, **p<0.01, ***p<0.001. Con, Control; I, immune checkpoint inhibitor αPD-1; V, FlaB-Vax; P, TLIF-PTT; PV, TLIF-PTT+FlaB Vax; PVI, TLIF-PTT+FlaB-Vax+αPD-1.

Given that T cell exhaustion accompanies PD-1 expression and IFN-γ modulates PD-L1 expression in both immune and tumor cells,^24^ we speculated that the PD-1 blockade should enhance the therapeutic efficacy of the combination of TLIF-PTT and FlaB-Vax. We investigated whether a triple combination therapy comprizing anti-PD-1, TLIF-PTT, and FlaB-Vax could induce more sustained tumor suppression and better therapeutic outcomes in the orthotopic Her2/neu breast cancer model (figure 6A). Treatment with either FlaB-Vax or anti-PD-1 alone did not meaningfully affect the survival of tumor-bearing mice (figure 6B). The double combination of TLIF-PTT and FlaB-Vax significantly prolonged survival (***p<0.001, Control vs TLIF-PTT+FlaB Vax) but could not save the lives of animals in the long run. Significantly, triple combination treatment (TLIF-PTT+FlaB Vax + αPD-1) extended the lifespan of the mice, with all treated animals surviving up to 250 days (***p<0.001, Control vs TLIF-PTT+FlaB-Vax+ αPD-1) (figure 6B). These findings indicate that anti-PD-1 significantly potentiated the therapeutic efficacy of the TLIF-PTT/FlaB-Vax combination and long-lasting immune memory.

### The triple combination of targeted liposomal formulation (TLIF)-photothermal therapy (PTT), flagellin-adjuvanted peptide vaccine (FlaB-Vax), and anti-PD-1 induced enhanced antigen-specific cellular immunity and robust epitope expansion

To elucidate the mechanism underlying the long-term relapse-free survival achieved by the triple combination of TLIF-PTT, FlaB-Vax, and anti-PD1, we investigated antigen-specific immune responses on day 14 (figure 6A). We found that the triple combination therapy induced the highest Her2 (p66; TYVPANASL) peptide-specific dextramer-positive CD8^+^ cells (6.32±0.474%) (figure 6C). When compared with the double combination (TLIF-PTT+FlaB Vax), a significantly higher number of dextramer-positive CD8^+^ T cells were detected in the triple combination group (**p<0.01, TLIF-PTT+FlaB Vax vs TLIF-PTT+FlaB Vax + αPD-1). And significantly higher numbers of Her2-specific IFN-γ producing spleen cells were detected in both double and triple combination therapies compared with other groups, while statistical significance was not observed between the two groups (***p<0.001, Control vs TLIF-PTT+FlaB Vax; ***p<0.001, Control vs TLIF-PTT+FlaB Vax + αPD-1) (figure 6D).

Given that the combination approach achieved better therapeutic outcomes and metastasis suppression, we hypothesized that TAs released from ICD-induced dying cancer cells should have induced epitope expansion. Though CD8^+^ cytotoxic T cells appeared to play a dominant role in tumor suppression, it was challenging to directly show T cell epitope expansion. As indirect evidence that the combinatorial approach had expanded the epitope repertoire for protective immune responses, we observed antibody responses to cancer cell antigens employing Western blotting and Fluorescence-Activated Cell Sorting (FACs) analysis using pooled sera collected on day 14. DD-Her2/neu cell lysate was subjected to SDS-PAGE and immunoblotting for Western blot analyses. For FACs analysis, DD-Her2/neu cells were surface stained with the collected pooled sera. Serum from naive mice was used as a negative control. Figure 6E and F show that the triple combination sera exhibited expanded antibody generation against the cancer cell lysate antigens and strongest surface-binding signals compared with mono- and double treatments. These results show that the combination of TLIF-PTT and FlaB-Vax expanded the epitope repertoire, which was further potentiated by the addition of anti-PD-1 therapy.

### The triple combination of targeted liposomal formulation (TLIF)-photothermal therapy (PTT), flagellin-adjuvanted peptide vaccine (FlaB-Vax), and anti-PD-1 generated very long-lasting immune memory

**Figure 7:**
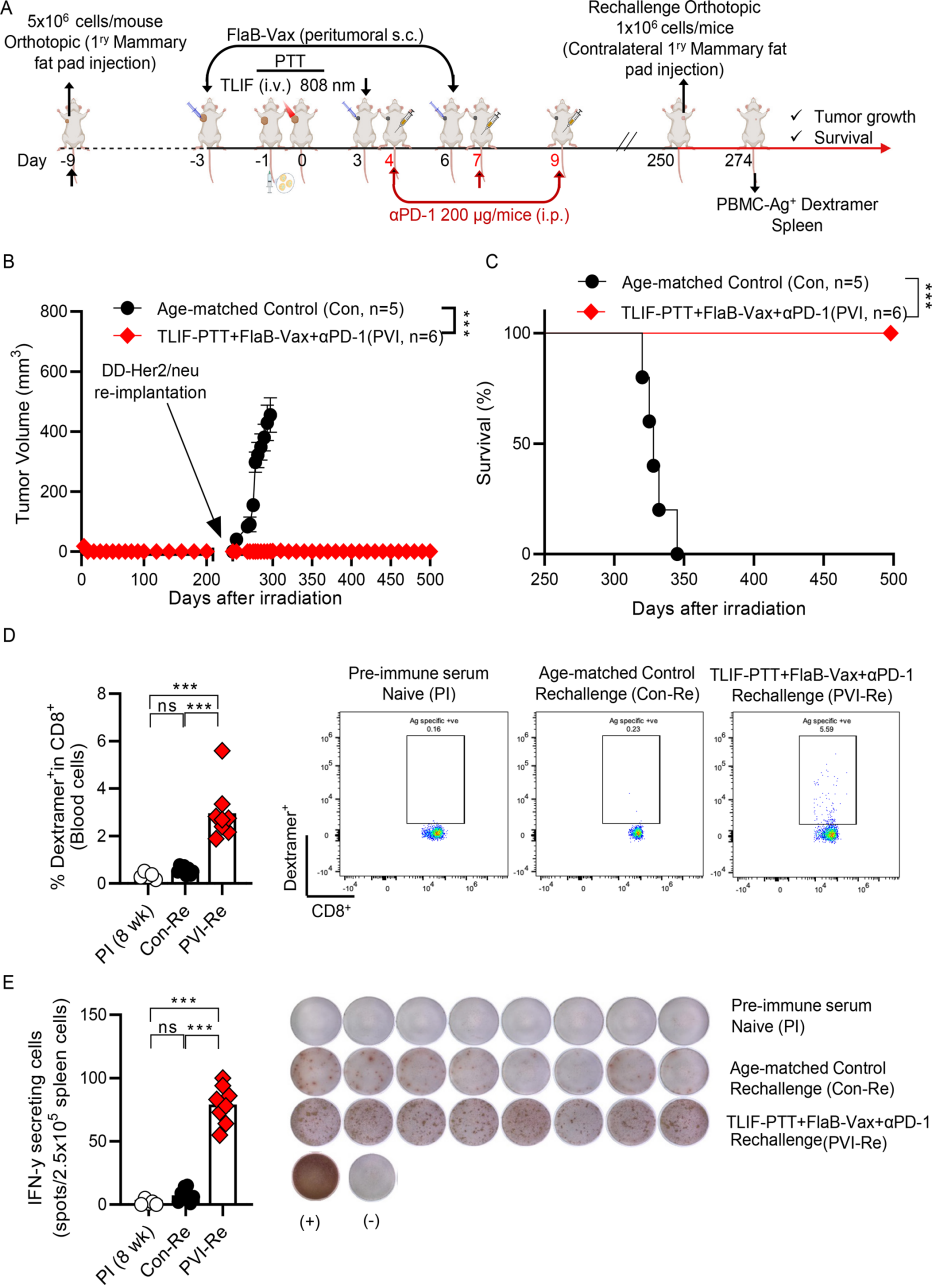
The triple combination of targeted liposomal formulation (TLIF)-photothermal therapy (PTT), flagellin-adjuvanted peptide vaccine (FlaB-Vax), and anti-PD-1 generated very long-lasting immune memory. (A) Schematic representation of the TLIF-PTT, FlaB-Vax, and anti-PD1 triple combination therapy and rechallenge schedules in a DD-Her2/neu breast orthotopic mouse model. After 250 days from the initial DD-Her2/neu cell implantation, mice with tumor eradication were rechallenged by injecting DD-Her2/neu cells into the contralateral mammary gland. Age-matched naive mice served as controls. (B) Growth kinetics of tumor growth in a DD-Her2/neu re-challenged breast orthotopic mice model after (n=5 or 6). (C) Survival proportion of mice in a DD-Her2/neu re-challenged breast orthotopic mice model after (n=5 or 6). The log-rank (Mantel-Cox) test was employed to compare the survival distributions between the two groups. (D) Determination of Her2 CTL epitope (p66; TYVPANASL)-specific dextramer-positive cells in peripheral blood CD8^+^ cells assessed by flow cytometry (n=5 or 8) in DD-Her2/neu re-challenged mice. (E) ELISpot analysis of IFN-γ-secreting cells in spleen cells stimulated with Her2 peptide (n=5 or 8, 2.5×10^5^ cells per well) in DD-Her2/neu re-challenged mice. Data are presented as the mean±SEM. Statistical significance was determined using the log-rank (Mantel-Cox) test, ordinary one-way analysis of variance, and Tukey’s multiple comparison test (survival, tumor growth, in vitro). Statistical significance is defined as follows: ns p>0.05 (ns, non-significance), *p<0.05, **p<0.01, ***p<0.001. PI, Pre-immune serum; Con, age-matched naive mice control; PVI+Re, TLIF-PTT+FlaB-Vax+αPD-1+Rechallenge.

To see whether effective immune memory was generated in tumor-eradicated animals treated with the triple combination (TLIF-PTT+FlaB-Vax+anti-PD-1), we re-implanted DD-Her2/neu cells to the contralateral mammary fat pad on day 250 (figure 7A). Age-matched naive mice implanted with the same number of DD-Her2/neu cells served as controls. Remarkably, compared with the control group, the tumor-eradicated surviving mice (PTT+FlaB Vax + anti-PD-1) showed no signs of tumor growth and achieved 100% survival, even on day 500. (figure 7B and C). This confirms that the triple combination treatment induced durable anti-tumor immunity, preventing cancer recurrence. We measured Her2-specific CD8^+^ T cells in the peripheral blood using dextramer staining 2 weeks after the rechallenge to assess the specificity of anti-tumor immune responses. Figure 7D and E show significantly increased Her2-specific dextramer-positive CD8^+^ T cells and IFN-γ-producing cells in the spleen in the treated mice (tumor-free mice after triple combination treatment). These results indicate that combining TLIF-PTT+FlaB Vax with anti-PD-1 leads to very long-lasting antigen-specific CD8^+^ memory T cells maintaining IFN-γ-producing ability.

## Discussion

Over the last two decades, immunotherapy has become the mainstream of cancer treatments. A significant breakthrough in cancer immunotherapy was the discovery of immune checkpoints that cancer cells take advantage of, deceiving the host immune system. The immunotherapy employing ICIs has drastically changed the cancer treatment landscape, especially for metastatic solid tumors, previously regarded to be incurable. ICIs mechanistically release the inhibitory brakes of T cells responsible for tumor suppression. ICI-activated immune responses operate within the TME and in the draining lymph nodes and distant lymphoid organs. However, the response rate to ICI varies among different types of cancers and ranges between 20% and 40%.^41^ The response rate would even go lower as the treatment cases accumulate. One cross-sectional study using publicly reported cancer statistics reported that only 12.5% were supposed to benefit.^42^ Combinatorial approaches employing ICI and other modalities have been studied to overcome this limitation in efficacy. Combining agents with different action mechanisms is expected to defeat multiple resistance mechanisms in targeted cancers.^43^ Based on this background, the present study was designed to find a new combinatorial immunotherapeutic strategy against intractable cancers, offering a promising outlook for future cancer treatments.

The so-called “cold” tumors non-responsive to ICI therapies exhibit immunosuppressive TME: the multimodal nature encompasses suppressive cytokines, lack of antigen presentation, apoptotic triggering of T cells, immune suppressive innate and adaptive cells, hostile metabolic states and nutrient deprivation, skewed microbiome, etc.^43 44^ Reversal of the immunosuppressive TME is crucial for the success of cancer immunotherapy.^33^ Tumor cells significantly contribute to the immunosuppressive TME, producing core factors that set the tissue environment to escape from host protective immune responses. In this regard, the reduction of tumor mass should be preceded or paralleled with immunotherapeutic treatments to get better outcomes. PTT would be an efficient way of debulking tumor mass if we could have appropriate photosensitizers preferentially accumulated in the TME. Another advantage of PTT is its ability to induce ICD. In a previous study, we reported a TLR5 ligand FlaB surface-conjugated nanoparticle encapsulating photo absorber IR-780 could induce ICD and modulate TME toward tumor-suppressive immune reactions by stimulating CD103^+^ migratory DCs.^26^ We observed that the TLR5 stimulating functionality was well preserved after the PTT. Flagellin is a resilient protein that maintains functionality under harsh physical conditions such as high temperature, acidity, and ROS.^26^ To make the PTT-modulated TME hotter, we introduced a tumor-specific peptide vaccine adjuvanted with thermostable FlaB in the present study. By employing vaccines targeting antigens specifically expressed by cancer cells, active immunosurveillance can be reactivated by increasing immune cell infiltration in TME (making it “hot”).^42^ There have long been worries about metastasis following tumor debulking by surgery or radiation.^43 44^ The tissue repair reaction and radiation-induced mutation following the tumor debulking were suggested to erroneously prompt metastasis. Cancer vaccine would counteract debulking-induced metastasis risk. In this context, we have selected the mouse DD-Her2/neu breast cancer model to evaluate the efficacy of the PTT-FlaB-Vax combination in inducing locoregional and systemic/abscopal immune responses that suppress recurrence and metastasis.

Among the diverse array of TLR ligands evaluated as adjuvants for cancer vaccines, flagellin, the cognate ligand for TLR5, has shown significant efficacy when co-formulated with peptide or protein antigens. We have demonstrated that bacterial flagellin from *Vibrio vulnificus*, known as FlaB, enhances antitumor immunity induced by TA through robust stimulation of TLR5 on antigen-presenting cells. FlaB operates a positive feedback circle in the TLR5 expression on DCs in the dLNs, which results in enhanced antigen-specific CD8^+^ cytotoxic T cell activity and IFN-γ production.^24 33^ Unlike other TLRs, TLR5 functionality is well maintained during immunosenescence, making it a potent immune adjuvant for vaccines against diseases prevalent in the elderly, such as cancers and Alzheimer’s disease.^32 45^ This study used FlaB as the adjuvant for in situ-generated TAs released by PTT and Her2 peptide antigen. Peritumorally administered FlaB in the first vaccine (FlaB-Vax) at day −3 should have primed antigen-presenting cells, both TME and dLN, which should have further boosted local and abscopal immune responses to both Her2 and PTT-ICD generated TAs. However, the combination of PTT and FlaB-Vax was not efficacious enough to save tumor-bearing animals’ lives (figures3  5). Interestingly, lung metastasis was significantly inhibited by the systemic immune responses induced by vaccination or PTT, while the combination of both was significantly more efficacious in extending survival (figure 5). Astonishingly, the addition of anti-PD-1 further enhanced tumor suppression and saved all the lives of treated animals (figure 6B). We started the anti-PD-1 therapy 1 day after the second vaccination so as not to induce improper T cell activation. It is known that prematurely activated T cells are non-responsive to anti-PD-1 treatment.^46^ Since the tumor was implanted at −9 days, and two-time vaccination and PTT were given to tumor-bearing mice, it was supposed that a sufficient T cell population (destined to be invigorated by anti-PD-1) should have been generated. Given the immune responses in Balb/c mice are rather predilected toward humoral immunity and Th2 responses,^47 48^ we may expect more robust tumor eradication in subjects whose immune system is more balanced towards cellular immunity (such as C57BL/6 mouse). More importantly, a significantly sustained immune memory was noted in the surviving mice even 500 days after the treatment. Based on our experimental data, we propose that the triple combination therapeutic strategy will synergistically save the lives of metastasis-prone cancer patients.

## Conclusion

This study underscores the potential of combining PTT with a therapeutic cancer vaccine adjuvanted with TLR5 ligand and an ICI (TLIF-PTT+FlaB Vax + αPD-1) as a potent therapeutic strategy for DD-Her2/neu tumors. Our findings indicate that this combination therapy can effectively address inadequate recruitment of T cells and upregulated expression of PD-L1 in tumors post-PTT, resulting in superior tumor control and complete remission rates. The demonstrated relapse-free survival of over 500 days in orthotopic and bilateral mice models highlights the promising efficacy of this approach.

## supplementary material

10.1136/jitc-2024-010272 online supplemental file 1

10.1136/jitc-2024-010272 online supplemental file 2

## Data Availability

Data are available upon reasonable request.
