# Supplementary material for: Thermoresistant flagellin-adjuvanted cancer vaccine combined with photothermal therapy synergizes with anti-PD-1 treatment
Source: J Immunother Cancer. 2025 Mar 20;13(3):e010272. doi: 10.1136/jitc-2024-010272 (PMC11931959; doi:10.1136/jitc-2024-010272)
Supplement: online supplemental file 1 [file jitc-13-3-s001.docx]

**Supplementary Materials and Methods**

**Synthesis and characterization of cRGD liposomes encapsulating ICG and FlaB (cRGD-lipo-ICG-FlaB; TLIF) nanoparticle.**

The cRGD liposomes encapsulating ICG and FlaB (TLIF, Figure 1A) were synthesized using the thin-film hydration method. Initially, cRGD liposomes were prepared as previously described (1) . Briefly, The cysteine-terminated cRGD peptide (C*GRGDSPK*) was conjugated to 1,2-distearoyl-sn-glycero-3-phosphoethanolamine-N- [maleimide (polyethyleneglycol)-2000] ammonium salt (DSPE-PEG-Mal, Avanti Polar Lipid, Alabama, USA) via thiol-maleimide coupling. The resulting conjugate was subsequently purified, lyophilized, and stored until further use. For liposome preparation, a lipid mixture containing dipalmitoyl-sn-glycero-3-phosphocholine (DPPC; Avanti Polar Lipids, Inc., Alabaster, AL, USA), cholesterol (Avanti Polar Lipids, Inc., Alabaster, AL, USA), and DSPE-PEG-Mal-conjugated cRGD was dissolved in an appropriate organic solvent in a 1.5:1.5:1 weight ratio. This mixture was transferred to a vial and dried under a vacuum to form a thin lipid film. Next, the lipid film was hydrated with an aqueous solution containing indocyanine green (ICG) by incubating at 60 °C for 10 minutes, forming heterogeneous, multivesicular liposomes. Following liposome formation, *Vibrio vulnificus* FlaB, an immunomodulator the TLR5 ligand adjuvant, was incorporated into the lipid mixture. The mixture underwent multiple freeze-thaw cycles (7 times) to ensure homogeneous integration of FlaB. Subsequently, the preparation was vortexed and sonicated under ice-cold conditions for 7 minutes to achieve a uniform liposomal suspension. The resultant solution was then passed through a 200 nm polycarbonate filter fixed in an Avanti mini extruder (Avanti Polar Lipids, Inc., Alabaster, AL, USA). This process resulted in the formation of cRGD-lipoICG-FlaB (TLIF) liposomes. The non-incorporated remaining components were subsequently removed via dialysis against water using a dialysis membrane with a molecular weight cutoff of 6-8 kDa. The liposome's hydrodynamic size and zeta potential were determined using dynamic light scattering (DLS) with a Zetasizer Nano Z (Malvern Instruments, Malvern, UK). The morphology of the nanoparticles was examined by field-emission transmission electron microscopy (FE-TEM) using a JEM-2100F (JEOL, Tokyo, Japan). The encapsulation efficiency of ICG and FlaB within the liposomes was assessed quantitatively. The concentration of ICG was determined by measuring its UV absorbance at the characteristic wavelength (771 nm) using a UV-2700 spectrophotometer (Shimadzu, Tokyo, Japan). The integrity of FlaB encapsulation in TLIF was confirmed by SDS-PAGE analysis followed by Western blotting, utilizing an anti-FlaB antibody (2-5). The TLR5-stimulating activity of the TLIF nanoparticles was validated as previously reported (6). Briefly, human embryonic kidney (HEK) 293T cells transfected with p3XFlag-hTLR5 and pNF-κB-Luc were incubated with various concentrations of TLIF for 24 hours post-transfection. For the TLIF + NIR irradiation group, cells were irradiated at 808 nm for 5 minutes prior to incubation with the transfected cells. After 24 h of incubation, the cells were lysed using lysis buffer, and luciferase activity was quantified using a luminometer (MicroLumat-Plus LB 96 V, Berthold, Wildbad, Germany). Luciferase activity was normalized to that of the control expression plasmid, pCMV-β-Gal (BD Biosciences Clontech, CA, USA).

**Determination of cytotoxic effects induced by TLIF treatment in DD-Her2/neu cells**

The cytotoxic effects of TLIF on the DD-Her2/neu cell line were assessed in pre- and post-laser irradiation conditions. Briefly, 1 × 10^4^ DD-Her2/neu cells were seeded into individual wells of a 96-well plate and incubated at 37 °C with 5% CO_2_ for 16 hours. The liposome suspensions, resuspended in cell medium, were subsequently added at various concentrations after the original cell medium was removed. After 4 hr incubation of DD-Her2/neu cells with the liposome the wells were exposed to laser (808 nm) irradiation for 5 minutes at 2 W/cm^2^ (PSU-III-LED, Changchun New Industries Optoelectronics, China) for laser treatment. After further incubation for 24 hours, the cell viability profile was quantified using the water-soluble tetrazolium salts assay (CellVia, Abfrontier, Korea) according to the manufacturer's protocol.

**ICG and FlaB release from TLIF nanoparticle**

To study the release of ICG from TLIF, the drug release study conducted in 10ml of 0.2% Tween 80. Given the hydrophobicity of ICG, 0.2% Tween 80 should be added to be quantitatively assayed. The absorbance of ICG were measured by UV–Vis spectrophotometry (UV-2700 Shimadzu, Tokyo, Japan). The dialysis method (MWCO 100 KDa) was employed to measure the drug release in 10 mL of release medium (1 × PBS). Both the samples medium were incubated in the dark with shacking at 200 rpm (7). At certain intervals, 1 mL of the medium was collected and substituted with an equal volume of fresh medium. The concentration of FlaB released from the TLIF was then quantified by bicinchoninic acid (BCA) assay.

**Photostability of cRGD liposomes encapsulating ICG and FlaB (cRGD-lipo-ICG-FlaB; TLIF) nanoparticle.**

The temperature curves of different concentrations of TLIF (5, 10, 20, 40 µg/mL) with 808 nm laser (2 W/cm^2^) were recorded at different time points with a thermal infrared imager (FLIR C3-X, USA). The temperature curves of TLIF (10 µg/mL) under different 808 nm laser powers (1, 1.5, 2 W/cm^2^) were also evaluated. The temperature changes of TLIF under six “5 second on-5 second off” cycles of NIR irradiation for were observed to assess he photothermal stability

**ICD evaluation *in vitro***

DDHer2/neu cells were seeded in 8 well cell culture plates (1 × 105 cells/well) for overnight growth. Based on ICG concentration, ICG (30 µg) or TLIF (30 µg) was added and incubated for 4 h. The laser treatment groups were irradiated with 808 nm laser (2 W/cm^2^, 5 min) and further incubated for 14 h. Subsequently, they were incubated with antibodies against calreticulin (CRT) (D3E6) (Abcam; ab 92516, UK) or high mobility group box 1 (HMGB1) (D3E5) (Cell Signaling; lot No. 4, USA) for 12 h at 4 °C, followed by secondary antibodies Alexa flour 488 (Invitrogen; 2380031, USA) or Alexa flour 546 (Invitrogen; lot no 2701068, USA). The cells were fixed with 4% paraformaldehyde for 20 min, permeabilized with 0.5% Triton X-100 for 10 min, and subsequently blocked with BSA for 1 h. Then, those cells were incubated with the primary antibody (anti-CRT from or anti-HMGB1 diluted 1:200) at room temperature for 2 h and next with the fluorescent-labeled secondary antibody (1:200) at room temperature for 1 h. Finally, the fluorescence images were taken with an inverted fluorescence microscope after DAPI staining.

**Biodistribution assessment of TLIF in a DD-Her22/neu tumor-bearing mice model**

A total of 5 x 10^6^ DD-Her2/neu cells suspended in 100 µl of PBS were injected subcutaneously into the right flank of each mouse. When the tumors had grown to a volume of approximately 80-100 mm³, the DD-Her2/neu tumor-bearing mice were administered intravenous injections of cRGD-lipoICG-FlaB (TLIF) or an ICG solution (with a dosage of 2 mg/kg of ICG). The accumulation of nanoparticles was tracked by measuring the fluorescence signal intensity at three-hour intervals. At 24 hours after administration, major organs, including the tumor, lungs, liver, kidneys, spleen, heart, brain, and lymph nodes, were subjected to fluorescence imaging**.** Photon signals were recorded using a fluorescence-labeled organism bioimaging instrument (FOBI; NeoScience Company, Korea). Quantitative analysis of the signal intensities in the specimens was performed by measuring the maximum photons per second per square centimeter per steradian (p/s/cm²/sr).

**Optimization of photothermal therapy (TLIF-PTT) in an orthotopic DD-Her2/neu breast cancer model**

To establish an orthotopic breast cancer model, 6-8 weeks old female BALB/c mice (Orient Bio Inc., Seongnam-si, South Korea) were subcutaneously injected with DD-Her2/neu (5.0 × 10^6^) cells into the right mammary fat pad to mimic human breast cancer (Figure 2A) closely. When the tumors reached the diameter of 3-5 mm, the mice were divided into several treatment groups. These included a control group (non-treated) and experimental groups treated with either cRGD-lipoICG-FlaB (TLIF) or lipoICG (LI). PTT treatment initiation was designated as day 0 across all experiments and depicted in all relevant figures. For PTT treatment, mice were intravenously infected with TLIF or LI formulation at an equivalent dose of 5 mg/kg ICG) solution. Subsequent laser irradiation was performed 24 hours after injection, using an 808 nm wavelength laser at a power density of 2 W/cm², maintained for 5 minutes at target temperatures of 40°C, 45°C, and 50°C. Tumor volume was assessed at three-day intervals throughout the experimental period. The tumor volume (V) was calculated using the formula *V = (tumor length) × (tumor width) × (tumor height) /* 2. Upon reaching a volume of 2,000 mm³, mice were euthanized to prevent undue suffering and maintain ethical standards following the study's protocol.

**Triple combination of TLIF-PTT, FlaB-Vax, and αPD-1 in an orthotopic DD-Her2/neu breast cancer model**

To test triple combination therapy of TLIF-PTT, FlaB-Vax, and αPD-1, we used an orthotopic DD-Her2/neu breast cancer model (Figure 6A). The orthotopic tumor-bearing mouse model was established by subcutaneously injecting 5.0 × 10^6^ DD-Her2/neu cells into the right mammary fat pad of female BALB/c mice aged six weeks old (Orient Bio Inc., Seongnam-si, South Korea). When the tumors reached the diameter of 3-5 mm, mice were divided into various treatment groups, including those non-treated (Control), αPD-1(αPD-1(I)), FlaB-Vax (V), TLIF-PTT (P), the combination of TLIF-PTT and FlaB-Vax (PV), and the triple combination of TLIF-PTT, FlaB-Vax, and αPD-1 (PVI). In the Vax, TLIF-PTT+FlaB-Vax, and TLIF-PTT+FlaB-Vax+αPD-1 treatment groups, the vaccine was administered peritumorally. Each dose of the vaccine comprised 4 µg of FlaB and 100 µg of the Her2 peptide (p66; TYVPANASL). Vaccination was performed 3 days before the start of PTT and continued on days 3 and 6 after PTT. The TLIF-PTT, TLIF-PTT+FlaB-Vax, and TLIF-PTT+FlaB-Vax+αPD-1 groups received intravenous injections of TLIF formulation at an equivalent dose of 5 mg/kg ICG. This administration was done 1 day before laser irradiation. The TLIF-PTT, TLIF-PTT+FlaB-Vax, and TLIF-PTT+FlaB-Vax+αPD-1 groups then underwent light irradiation at 808 nm wavelength and a power density of 2 W/cm², maintained at 50°C for 5 minutes, precisely 24 hours post-injection of nanoparticles. Tumor volume was monitored at three-day intervals using the formula: *V = (tumor length × tumor width × tumor height)* / 2. Ethical euthanasia was performed upon reaching a tumor volume of 1500 mm³. In experiments exploring combination therapies with immune checkpoint inhibitors (ICIs), anti-PD1 (clone RMP1-14, BioXCell, Lebanon, USA) was administered intraperitoneally (i.p.) at a dosage of 200 µg per dose on days +4, +7, and +9 following photothermal therapy (PTT). Tumor volume was measured at three-day intervals using the formula *V = (tumor length) × (tumor width) × (tumor height)* / 2. Ethical euthanasia was performed upon reaching a tumor volume of 2,000 mm³.

**Detection of Her2-specific CD8^+^ T cells in the blood**

To assess Her2-specific CD8+ T cells, peripheral blood samples were collected from mice and stained with PE-conjugated T-Select H-2Kd HER2 Tetramer-TYLPTNASL reagent (MBL, Life Sciences, Code No. TS-M526-1, Japan) or PE-conjugated MHC Dextramer H-2 Kd TYLPTNASL Her2/neu 63-71 (Immudex, Code No. JE3296-F, USA) following the manufacturer's instructions. Tetramer and Dextramer staining were combined with surface staining using APC-conjugated anti-mouse CD8α (BioLegend, Cat # 100712, Clone: 53-6.7) and analyzed by flow cytometry.

**Detection of Her2-specific IFN-γ production using ELISpot analysis**

Single-cell suspensions from the spleen (SPL), tumor-draining lymph nodes (TDLNs), and auxiliary lymph nodes (aLN) were prepared. A total of 2.5 × 10^5^ SPL cells or 1.5 × 10^5^ TDLNs or aLNs cells were seeded into 96-well Filtration ELISpot plates (Merck, HAMAS4510) and stimulated with 10 μg/ml of the Her2 CTL short peptide (p66; TYVPANASL). Cells stimulated with 10 ng/ml concanavalin A were positive controls. After two days of culture for SPL cells or five days for TDLNs and aLNs cells, IFN-γ-producing cells were detected using a mouse IFN-γ ELISpot Set (BD Biosciences, 551083) following the manufacturer's instructions. The spots were developed using an AEC substrate set (BD, 551951). IFN-γ-producing cells were analyzed using a CTL-ImmunoSpot Analyzer and ImmunoSpot Professional Software version 5.0 (Cellular Technology, Shaker Heights, OH, USA).

**Detection of therapy-mediated antibody responses in the blood**

DD-Her2/neu cell lysate was subjected to SDS-PAGE gel electrophoresis and transferred to nitrocellulose membranes. The membranes were then blocked for 4 hours at 25°C and stained overnight at 4 °C with serum from treated mice and mouse anti-β-actin. After washing, the membranes were stained with goat anti-mouse immunoglobulin/HRP and goat anti-rabbit immunoglobulin/HRP for 1 hour and 30 minutes at 25°C. Imaging was conducted using an Odyssey scanner (Licor). The indicated tumor cells were incubated with 5% serum collected on day 14 after PTT. The cells were washed, stained with an APC-conjugated mouse-IgG-specific secondary antibody, and analyzed by flow cytometry (Invitrogen, Cat# A-865).

**References**

1. Sun EG, Vijayan V, Park M-R, Yoo K, Cho S-H, Kyun W, et al. Suppression of triple-negative breast cancer aggressiveness by LGALS3BP via inhibition of the TNF-α–TAK1–MMP9 axis. Cell Death Discovery. 2023;9.

2. Khim K, Puth S, Radhakrishnan K, Nguyen TD, Lee YS, Jung CH, et al. Deglycosylation of eukaryotic-expressed flagellin restores adjuvanticity. NPJ Vaccines. 2023;8(1):139.

3. Rhee JH, Khim K, Puth S, Choi Y, Lee SE. Deimmunization of flagellin adjuvant for clinical application. Curr Opin Virol. 2023;60:101330.

4. Zheng JH, Nguyen VH, Jiang S-N, Park S-H, Tan W, Hong SH, et al. Two-step enhanced cancer immunotherapy with engineered <i>Salmonella typhimurium</i> secreting heterologous flagellin. Science Translational Medicine. 2017;9(376):eaak9537.

5. Hong SH, Byun Y-H, Nguyen CT, Kim SY, Seong BL, Park S, et al. Intranasal administration of a flagellin-adjuvanted inactivated influenza vaccine enhances mucosal immune responses to protect mice against lethal infection. Vaccine. 2012;30(2):466-74.

6. Tan W, Zheng JH, Duong T-MN, Koh Y-I, Lee SE, Rhee JH. A Fusion Protein of Derp2 Allergen and Flagellin Suppresses Experimental Allergic Asthma. Allergy Asthma Immunol Res. 2019;11(2):254-66.

7. Yi H, Lu W, Liu F, Zhang G, Xie F, Liu W, et al. ROS-responsive liposomes with NIR light-triggered doxorubicin release for combinatorial therapy of breast cancer. Journal of Nanobiotechnology. 2021;19(1):134.

**
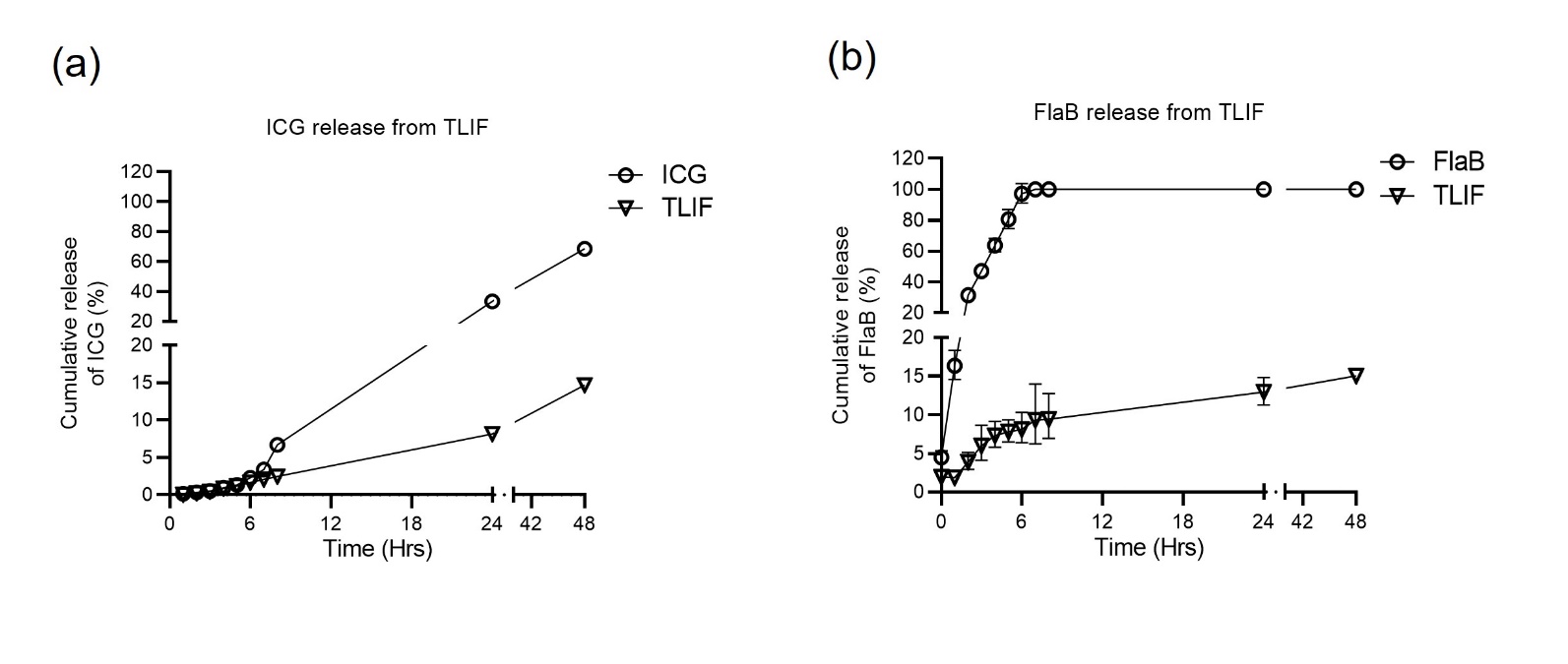
Supplementary Figures**

**Figure S1. Drug release study.** (a) ICG release from TLIF, UV-vis NIR spectra of non-encapsulated free ICG and ICG from TLIF in PBS containing 0.2% Tween 80. (b) FlaB release from TLIF, cumulative release of non-encapsulated free FlaB and FlaB from TLIF in PBS.


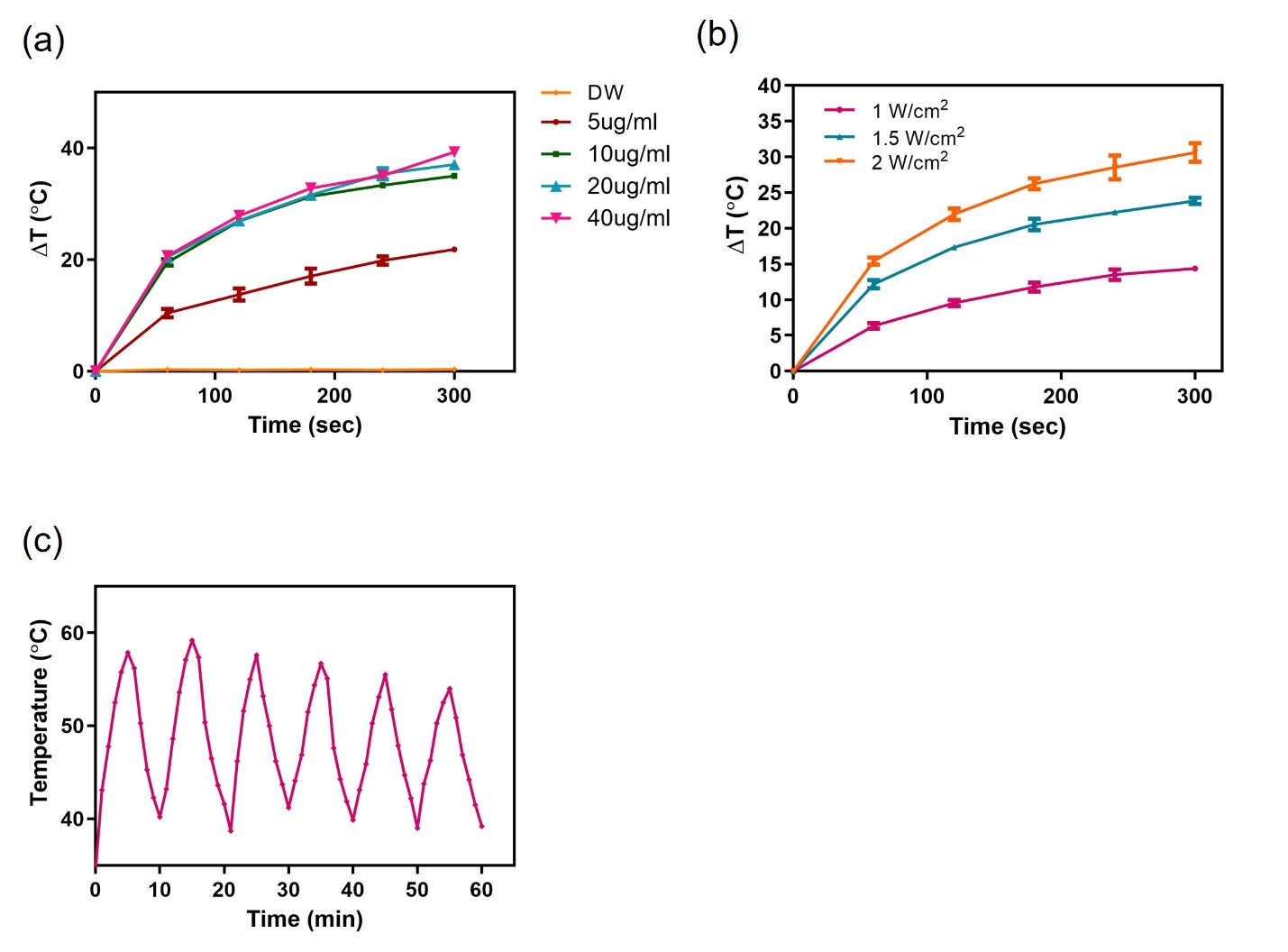


**Figure S2.** **Photostability of cRGD liposomes encapsulating ICG and FlaB (cRGD-lipo-ICG-FlaB; TLIF) nanoparticle** (a) Temperature rising profiles of TLIF with varying ICG concentrations (5, 10, 20, and 40 µg/mL) under 808 nm laser irradiation at 2 W/cm² for 5 minutes. (b) Temperature rising profiles of TLIF at different laser power (1, 1.5, and 2 W/cm²) with a fixed ICG concentration (10 µg/mL) under 808 nm laser irradiation. (c) Photothermal stability test of TLIF (10 µg/mL) by repeated heating and cooling cycles under continual 808 nm laser irradiation at 2 W/cm².

**
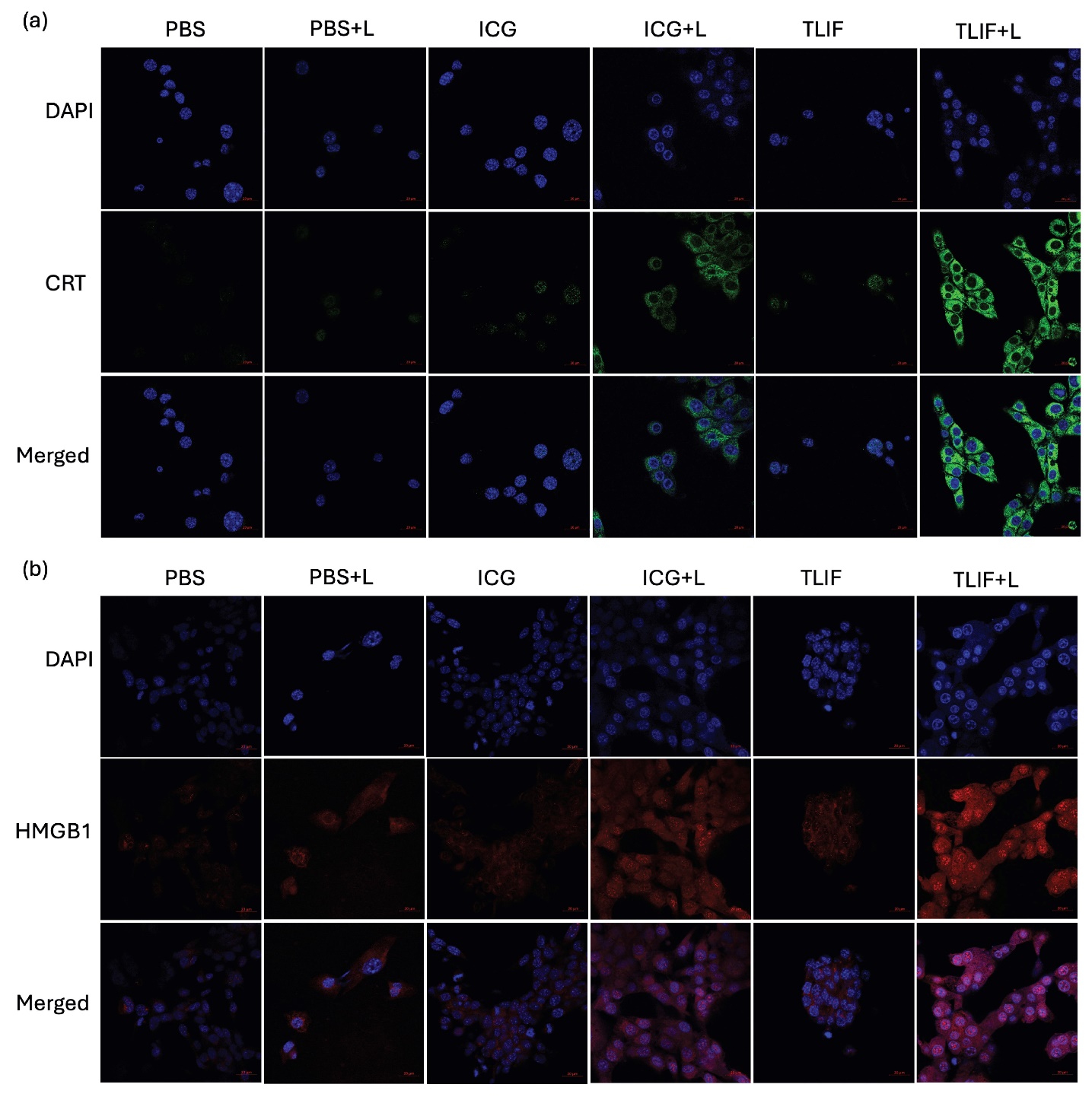
**

**Figure S3.** **Investigation of ICD evaluation in vitro.** Robust ICD was induced by NIR irradiation in cells cultured in the presence of TLIF. ICG was used as a comparator of TLIF. The expression of CRT (a) and HMGB1 (b) increased in DD Her2/neu cells co-incubated with free ICG or TLIF nanoparticles was significantly induced by the NIR irradiation. Scale bar: 20 µm.
