# Supplementary material for: Thermoresistant flagellin-adjuvanted cancer vaccine combined with photothermal therapy synergizes with anti-PD-1 treatment
Source: J Immunother Cancer. 2025 Mar 20;13(3):e010272. doi: 10.1136/jitc-2024-010272 (PMC11931959; doi:10.1136/jitc-2024-010272)
Supplement: online supplemental file 2 [file jitc-13-3-s002.pdf]

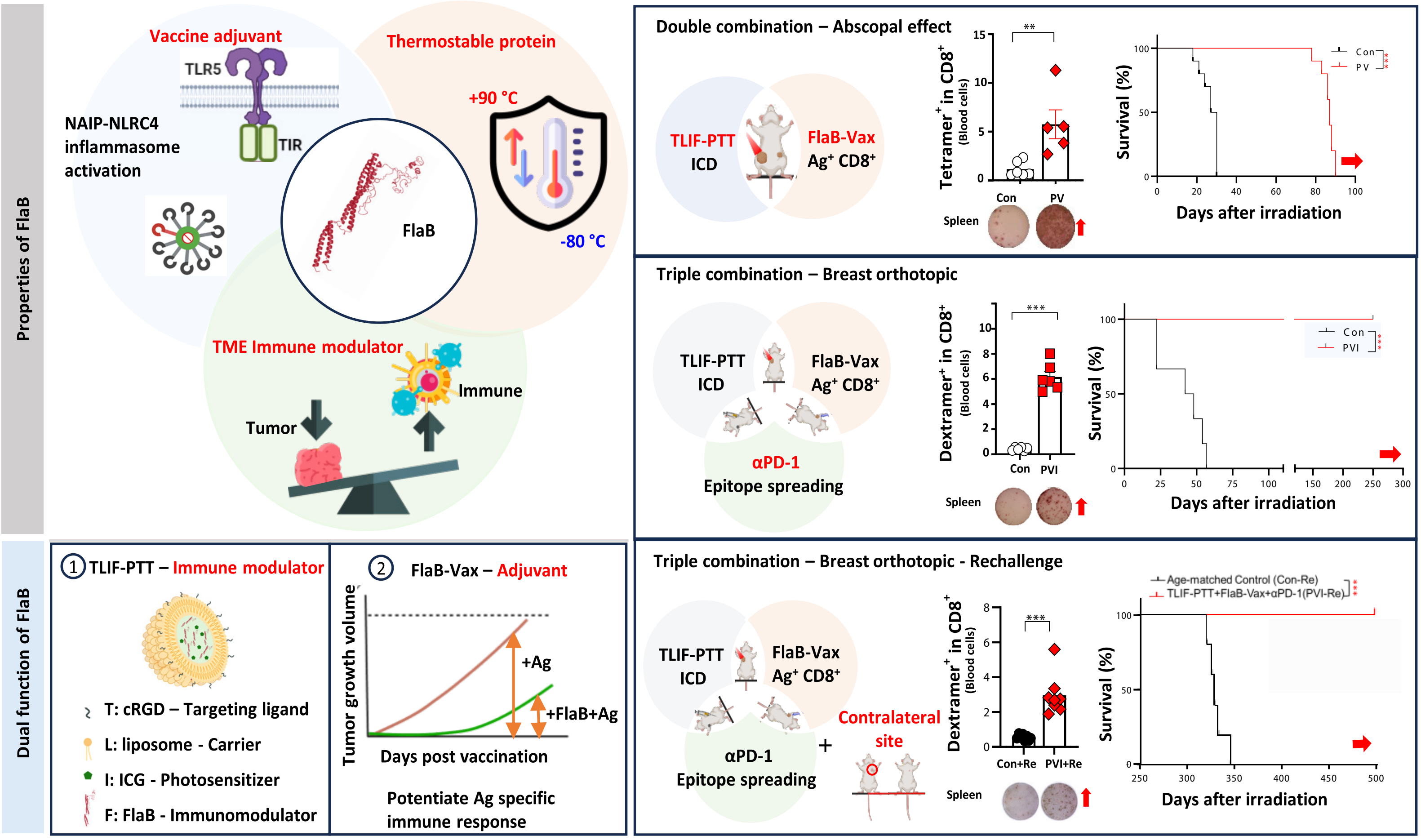

Properties of FlaB
Vaccine adjuvant
TLR5
TIR
NAIP-NLRC4 inflammasome activation
Thermostable protein
+90 °C
-80 °C
FlaB
TME Immune modulator
Immune
Tumor
Dual function of FlaB
① TLIF-PTT – Immune modulator
T: cRGD – Targeting ligand
L: liposome - Carrier
I: ICG - Photosensitizer
F: FlaB - Immunomodulator
② FlaB-Vax – Adjuvant
Tumor growth volume
+Ag
+FlaB+Ag
Days post vaccination
Potentiate Ag specific immune response
Double combination – Abscopal effect
TLIF-PTT ICD
FlaB-Vax Ag+ CD8+
Tetramer+ in CD8+ (Blood cells)
Con
PV
Spleen
Survival (%)
Days after irradiation
Triple combination – Breast orthotopic
TLIF-PTT ICD
FlaB-Vax Ag+ CD8+
αPD-1 Epitope spreading
Dextramer+ in CD8+ (Blood cells)
Con
PVI
Spleen
Survival (%)
Days after irradiation
Triple combination – Breast orthotopic - Rechallenge
TLIF-PTT ICD
FlaB-Vax Ag+ CD8+
αPD-1 Epitope spreading
Contralateral site
Dextramer+ in CD8+ (Blood cells)
Con+Re
PVI+Re
Spleen
Age-matched Control (Con-Re)
TLIF-PTT+FlaB-Vax+αPD-1(PVI-Re)
Survival (%)
Days after irradiation
